# A theory-based video intervention to enhance communication and engagement in online health communities: two experiments

**DOI:** 10.1080/21642850.2022.2032074

**Published:** 2022-02-09

**Authors:** Michael Kilb, Oliver Dickhäuser, Jutta Mata

**Affiliations:** a Department of Psychology, School of Social Sciences, University of Mannheim, Mannheim, Germany; b Mannheim Center for Data Science, University of Mannheim, Mannheim, Germany; c Center for Adaptive Rationality, Max Planck Institute for Human Development, Berlin, Germany

**Keywords:** Social networking sites, online communities, behavior change interventions, need-support, self-determination theory

## Abstract

**Background:**

Online communities and social networking sites have great potential for supporting health behavior change. However, interventions vary greatly in participants’ engagement rates and, consequently, their effectiveness. Theory-based interventions in real-world contexts are needed to further increase engagement and effectiveness.

**Methods:**

We experimentally tested whether a video intervention teaching Self-Determination-Theory-based communication strategies increases need-supportive communication strategy use over one week (Study 1, *N* = 76) and perceived need support, engagement, and goal attainment in a behavior change intervention supported by a forum-based online community (Study 2, *N* = 537). In Study 2, participants chose a goal (increasing either fruit or vegetable consumption or increasing moderate or vigorous physical activity) and joined an online community for 2 weeks. Data from both experiments were analyzed with mixed models and follow-up tests.

**Results:**

In Study 1, participants in the intervention but not in the control group showed an increase in the number of need-supportive communication strategies used both immediately and one week after the intervention (condition × time interaction, *partial η*^2^ = 0.31). In Study 2, participants who watched the intervention video had a higher number of postings and reported a higher subjective forum use frequency (but not a higher number of logins) compared to participants who watched the control video. However, the effect on the subjective forum visit frequency was not robust. There were no intervention effects on perceived need support, goal attainment, or secondary outcomes. The results might be explained by low application of need-supportive communication strategies.

**Conclusion:**

A brief video intervention may be a suitable, low-cost intervention to promote need-supportive communication strategy use, benefitting both engagement and behavior change. Future studies should incorporate additional means to further improve communication strategy uptake and engagement in online communities.

## 1. Introduction

### 1.1. Background

Lifestyle behaviors, such as physical inactivity or a poorly balanced diet, are the main risk factors for premature mortality and disability caused by noncommunicable diseases (GBD 2017 Diet Collaborators, 2019; GBD 2019 Risk Factors Collaborators, 2020; Lee et al., 2012). Worldwide, there is a high need for interventions tackling these lifestyle behaviors. An important medium for reaching a high proportion of the population with interventions is the internet. Across the globe, access to the internet has grown rapidly in the last decade (International Telecommunication Union, 2020; Roser, Ritchie, & Ortiz-Ospina, 2015). In 2020, the International Telecommunication Union estimated that in 2019, 51% of the world population used the internet – for example, 77% of individuals in the United States, 82% in Europe, and 29% in Africa (International Telecommunication Union, 2020). In 2020, 89% of the German population over age 14 used the internet occasionally, and 72% used it daily (Beisch & Schäfer, 2020). One attractive vehicle for delivering behavior change interventions on a large scale is online communities, such as those found on social networking sites, including Facebook, Instagram, Twitter, and Reddit. In 2021, most U.S. adults used social networking sites: 69% used Facebook, 40% Instagram, and 18% Reddit (Pew Research Center, 2021). In Germany in 2020, 37% of the population over 14 used Facebook and 25% Instagram (Beisch & Schäfer, 2020). Social networking sites provide the opportunity to connect people worldwide through person-to-person relationships or building online communities.

Social networking sites are increasingly being used for health behavior change interventions (Dahl, Hales, & Turner-McGrievy, 2016; Hou, Charlery, & Roberson, 2014; Maher, Ryan, Kernot, Podsiadly, & Keenihan, 2016; Petkovic et al., 2021), typically as a platform to disseminate intervention materials (e.g. Napolitano, Hayes, Bennett, Ives, & Foster, 2013) or as an add-on in multicomponent interventions to provide a supportive environment for behavior change (e.g. Godino et al., 2016). Because these platforms are widely integrated into daily social life, they allow to easily reach a wide range of populations with evidence-based health behavior change interventions (Arigo et al., 2019; Petkovic et al., 2021). They also likely increase the dose of effective ingredients of behavioral interventions (‘behavior change techniques’; Michie et al., 2013), as they are remotely and continuously accessible, and frequently used (Mata & Baumann, 2017). They also provide unique features for interacting with like-minded people in private online spaces such as private forums or Facebook groups, increasing social support and social influence (Dahl et al., 2016; Zhang et al., 2016). Findings are mixed but results of several meta-analyses suggest a small positive effect of interventions using social networking sites for health behavior change (Laranjo et al., 2015; Petkovic et al., 2021; Waring et al., 2018; Williams, Hamm, Shulhan, Vandermeer, & Hartling, 2014; Yang, 2017). Nevertheless, little is known about potential mechanisms underlying these effects because social networking sites’ unique effects are rarely isolated, and hypothesized intermediate outcomes are hardly ever tested (Waring et al., 2018; Yang, 2017). There are also some challenges when using online communities and social networking sites as health behavior change interventions, such as privacy concerns (Arigo et al., 2019; Klassen, Douglass, Brennan, Truby, & Lim, 2018), adapting intervention contents (Pagoto et al., 2016), or open questions regarding necessary levels of engagement and factors influencing engagement (Arigo et al., 2019; Miller et al., 2019; Short et al., 2018).

One of the biggest problems of these interventions is a typically low user engagement. There are different ways of measuring actual and perceived engagement with server data or self-report, such as the number of logins, the number of postings written, the number of posts viewed, or the number of provided reactions, which can be categorized into broader categories, e.g. frequency or intensity of engagement (Short et al., 2018). Engagement varies substantially between studies and typically declines over the intervention period (Arigo, Pagoto, Carter-Harris, Lillie, & Nebeker, 2018; Waring et al., 2018; Yang, 2017). Research shows that there is often a high number of non-users and passive users (lurkers), who do not or only rarely contribute to the interactions within online communities and social networking sites, and a low number of very active users (power users) who contribute most of the interactions (Carron-Arthur, Ali, Cunningham, & Griffiths, 2015; Edelmann, 2016; Hampton, Goulet, Marlow, & Rainie, 2012). However, even in the most extreme form of lurking (i.e. never posting or contributing to online communities and in social networking sites), lurkers might still profit from silently using the platform and viewing the content (Edelmann, 2016). Engagement is positively related to intervention outcomes: For example, frequency and number of postings as well as the number of reactions predict weight loss success in social-networking-site-based weight loss interventions (Pagoto et al., 2017; Xu & Cavallo, 2021). Still, the optimal kind (e.g. actively posting vs. passively viewing content) and amount of engagement for intervention success in digital health behavior interventions is often unclear and could depend on person and/ or intervention characteristics (Miller et al., 2019; Short et al., 2018). Furthermore, the effects of different forms of engagement in online communities and social networking sites likely depend on the amount and actual content of the other users’ postings and reactions (Xu & Cavallo, 2021). Since at least some level of engagement is necessary for intervention success, interventions should ensure that participants stay engaged through the intervention period. It should, however, be noted that in some cases sustained engagement may not be necessary. For example, when participants find effective behavior change techniques early in the intervention and can carry them out by themselves, thus, do not depend on the intervention context. Characteristics of a digital behavior change intervention, particularly the experience and interaction within online communities, can influence the engagement of participants (Perski, Blandford, West, & Michie, 2017). Providing a more supportive and helpful environment is a promising approach to increase participants’ engagement within online communities. Given that engagement is essential for positive intervention outcomes, strategies for increasing engagement may be at least as important as the intervention itself. One way to simultaneously increase engagement and successful behavior change in interventions involving online communities and social networking sites is to target interpersonal communication, one of their core features. Self-Determination Theory (SDT; Deci & Ryan, 2000; Ryan & Deci, 2017) can provide a theoretical framework to understand interpersonal communication in online communities.

### 1.2. Self-determination theory

SDT (Deci & Ryan, 2000; Ryan & Deci, 2017) is a well-established macrotheory on human motivation, according to which there are 3 basic psychological needs: need for autonomy, relatedness, and competence. The fulfillment of these needs is assumed to energize and foster human motivation (Deci & Ryan, 2000; Ryan & Deci, 2017), particularly a more autonomous form of motivation (Ng et al., 2012). Autonomous motivation is important for the long-term regulation of health behaviors (Kwasnicka, Dombrowski, White, & Sniehotta, 2016), such as eating a healthy diet (Teixeira, Patrick, & Mata, 2011). Findings from interventions suggest that changes in perceived need support are associated with changes in autonomous motivation and successful behavior change (Ntoumanis et al., 2021). Importantly, a person’s social environment can be need-supportive (often also referred to as autonomy supportive), thus supporting or undermining the fulfillment of the 3 basic psychological needs (Deci & Ryan, 2000; Ryan & Deci, 2017; Ryan, Patrick, Deci, & Williams, 2008).

In the last decade, efforts have been made to integrate motivational theories such as SDT and more traditional social cognition models such as the theory of planned behavior (TPB; Ajzen, 1991), a theory that has been successfully applied to predict health behavior (McEachan, Conner, Taylor, & Lawton, 2011, 2016). This theoretical integration suggests that a relatively more autonomous motivation may influence behavior at least partly via attitudes, subjective norms, and perceived behavioral control (Hagger & Chatzisarantis, 2009). A more refined version of the theory of planned behavior, the reasoned action approach (Fishbein & Ajzen, 2011), further differentiates different sub-facets of these three constructs, namely experiential and instrumental attitudes, descriptive and injunctive norms, capacity (related to self-efficacy) and autonomy (Fishbein & Ajzen, 2011; McEachan et al., 2016).

### 1.3. An SDT-view on interpersonal communication

Health behavior change interventions can be more or less need-supportive (Gillison, Rouse, Standage, Sebire, & Ryan, 2019; Ryan et al., 2008; Silva, Marques, & Teixeira, 2014); the interpersonal communication between users in digital social environments, the key feature of interventions involving online communities and social networking sites, therefore play a central role (e.g. Ntoumanis, Quested, Reeve, and Cheon (2017); Su and Reeve (2011) for findings in sports and teaching). Compared to increasing engagement, which focuses on the *quantity* of social interactions in online communities and social networking sites (e.g. number of logins, postings, or reactions), increasing need-supportive communication focuses on the style and *quality* of social interactions. Need-supportive communication has been described as an empathetic and patient rather than pressuring communication style (Ntoumanis et al., 2017). In need-supportive communication, one acknowledges others’ perspectives and feelings, provides meaningful rationales, offers choices, nurtures inner motivational resources, and uses noncontrolling language (Su & Reeve, 2011). Further, it is possible to learn to use a need-supportive communication style with others through training (Su & Reeve, 2011). Importantly, no such interventions have been developed and tested in the context of health behavior interventions involving online communities or social networking sites. This is an important lack of research as online communities are usually large, and interventions involving social networking sites that have the power to reach a wide range of people potentially are particularly promising.

### 1.4. Increasing need-supportive communication influences behavior change and engagement

Promoting a need-supportive communication style within online communities should contribute to need fulfillment and perceived need support, autonomous motivation, and successful behavior change (Gillison et al., 2019; Ntoumanis et al., 2021). Building on the integrated SDT-TPB model (Hagger & Chatzisarantis, 2009) and the advancement of the TPB to the reasoned action approach, we expect intervention effects on behavior change to be at least partially mediated by changes in autonomous (but not controlled) motivation (Ng et al., 2012; Ntoumanis et al., 2021), and by changes in attitudes, social norms, and self-efficacy (McEachan et al., 2016; Sheeran et al., 2016). Higher need fulfillment may further lead to higher perceived social support, one key mechanism of health behavior change, which is usually targeted with social-networking-site and online-community-based health behavior interventions (Dahl et al., 2016; Petkovic et al., 2021), because participants feel understood and supported. Increased need support could also positively impact engagement and participation within online communities by improving users’ experiences, and in consequence make interventions more successful (Perski et al., 2017; Waring et al., 2018).

### 1.5. Aims and overview

This paper describes 2 studies evaluating a brief SDT-based intervention with the goal of instructing participants about need-supportive communication strategies within online communities. In Study 1, we developed a brief intervention video and tested – in an experimental setting – how this intervention changed communication strategies. We expected an increased use of the six targeted SDT-based need-supportive communication strategies from baseline to follow-up in the intervention group, but not the control group. In Study 2, we tested the effects of the intervention video on goal attainment, perceived need support, and engagement (primary outcomes) in the context of a behavior change intervention supported by a forum-based online community. Additionally, we examined the effect of the intervention on the following secondary outcomes, which could explain a potential positive effect of the intervention on goal attainment: autonomous motivation, experiential and instrumental attitudes, self-efficacy, perceived descriptive and perceived injunctive norms, and perceived social support. For all outcomes, we expected positive effects of the intervention video, that is, higher values in the intervention group compared to the control group. Based on the existing literature, we did not expect a positive effect on controlled motivation.

## 2. Materials and methods

### 2.1. Design and procedure

In Study 1, participants were recruited online via the study management system of a German University as well as word-of-mouth recommendations. After providing baseline data, participants were given 3 fabricated Facebook postings that consisted of a problem description in the nutrition context. Participants were asked to write a response to each of the postings (pre-intervention time point). Subsequently, participants were randomized to watch either the intervention containing information about need-supportive communication strategies or the control video containing more general communication tips (see below for intervention development). Thereafter, they answered short questions about the video and received a written summary of the communication strategies to ensure that all participants remembered the strategies. They were then asked to respond to 3 similarly structured Facebook postings and to apply the newly learned communication strategies in their responses (post-intervention time point). After 1 week, participants responded to 3 additional Facebook postings, with the instruction to use the learned communication strategies, but without watching the video again (1-week follow-up time point). The study was conducted in accordance with the Declaration of Helsinki.

In Study 2, which was preregistered on the Open Science Framework (https://osf.io/x7uv2), participants were recruited in two recruitment phases via advertisements in offline and online media, as well as Facebook ads. The intervention content was coded using the behavior change technique (BCT) taxonomy v1 (Michie et al., 2013) and the motivation and behavior change techniques (MBCTs) taxonomy for interventions based on Self-Determination Theory (Teixeira et al., 2020). The study was advertised as a ‘health challenge’ to change one’s eating or physical activity behavior for 2 weeks while participating in a forum-based online community. To increase adherence and decrease dropout from the study, we gave participants the choice between 4 different goals: increase fruit intake, increase vegetable intake, increase moderate physical activity, or increase vigorous physical activity. Participants could first choose the behavioral domain (physical activity vs. eating fruits and vegetables) after which they could choose the more specific goal (see Appendix A). Participants were encouraged to choose a behavior which they do not show frequently and want to increase. All participants received the goal to increase intake or activity by 33% (BCT 1.1 Goal setting (behavior)). The behavioral target (number of portions or minutes of activity) was automatically calculated in the baseline assessment by using the individual baseline values of the selected behavior. After completing baseline questionnaires, participants were randomized to watch either the intervention or the control video. Next, participants were invited to join the forum-based online community that was established for their behavioral goal and intervention type where they could support each other (BCT 3.1. Social support (unspecified)). Appendix A contains an overview of all self-selected decisions and randomizations. The online communities were created exclusively for the study participants. A screenshot of one of the online communities can be seen in Appendix B. Participants subsequently worked on their goals for 2 weeks, after which they were asked to complete the follow-up questionnaire to assess primary and secondary outcome variables. Links to the videos and written summaries of the communication strategies were placed as community rules at the top of the forums and were accessible during the intervention period. One moderator monitored the postings in the forums over the intervention period to answer technical questions and to detect potential hostile postings. The moderator did not provide any other additional advice to participants. Participants were sent up to 2 reminder e-mails to answer the follow-up questionnaire. The study was approved by the Institutional Review Board of the University of Mannheim (11/2020).

### 2.2. Intervention development

**Figure 1.**
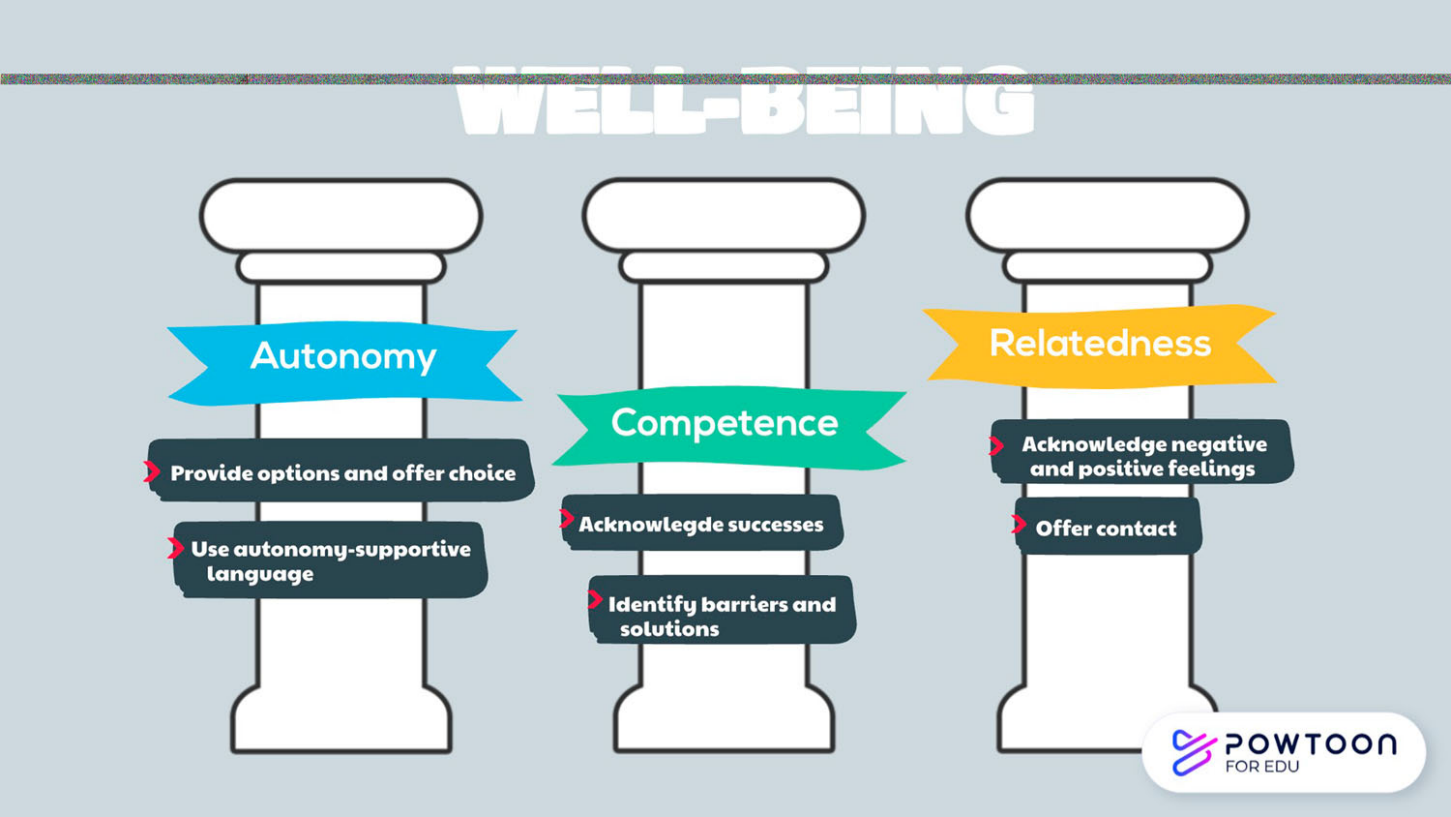
Screenshot of the intervention video with the communication strategies based on Self-Determination Theory (content translated from German to English).

We developed a brief educational video based on SDT (Deci & Ryan, 2000; Ryan & Deci, 2017) with 6 communication strategies to address need-supportive communication (see Figure 1). Additionally, we created a control video with general netiquette rules. Netiquette means general tips and rules for respectfully interacting in online communities. Typical rules vary by platforms or even by sub-forums (e.g. in Reddit, every sub-reddit can create own community guidelines). For our control video, we included key rules which focus on the understandability of postings and a non-hostile community environment but did not overlap with the SDT-based need-supportive communication strategies. The included rules were ‘stay on topic’, ‘adapt to target audience’, ‘be respectful’, ‘avoid ambiguities and abbreviations’, and ‘pay attention to spelling and grammar’. The final videos have a duration of about 3 min and were created with the software Powtoon. Both videos were designed to be as similar as possible and consisted of a short introduction to social networking sites’ and online communities’ advantages in supporting behavior change and goal attainment. Participants were then introduced to the different communication strategies, and the intervention video also contained brief information about the 3 basic psychological needs. Finally, both videos contained a written communication example with the application of the respective communication strategies (BCT 4.1 Instructions on how to perform the behavior). Potential communication strategies were derived from 2 meta-analyses on SDT-based interventions (Gillison et al., 2019; Su & Reeve, 2011). The first author and 5 master’s students (in psychology) discussed possible communication strategies and their applicability in the context of written online communication. Two strategies for each basic psychological need were determined by joint discussion (MBCT 3. Use noncontrolling, informational language; MBCT 6. Provide choice; MBCT 8. Acknowledge and respect perspectives and feelings; MBCT 13. Providing opportunities for ongoing support; MBCT 15. Address obstacles for change; MBCT 18. Offer constructive, clear, and relevant feedback).

### 2.3. Measures

#### 2.3.1. Study 1

Demographic characteristics included age, gender, and highest educational attainment. Educational attainment level was subsequently coded according to the International Standard Classification of Education (ISCED; UNESCO Institute for Statistics, 2012) and recommendations by Eurostat (Eurostat, 2021) as low (ISCED levels 0–2), medium (ISCED levels 3 and 4), and high (ISCED levels 5–8).

The first author and 5 Master’s students who were involved in the development of the video intervention developed a first version for a coding scheme through joint discussions. Two trained Bachelor students in psychology blind to the experimental condition coded 20 responses separately. Both raters and the first author discussed the results, resolved disagreements, and refined the coding scheme. Subsequently, both raters coded 29% (170/595) of the written responses (use of each of the 6 strategies; no = 0 vs. yes = 1) to estimate interrater reliability. Interrater agreement, calculated with ReCal OIR (Freelon, 2013), was adequate to good (percentage agreement = 76.5%–95.3%; Krippendorff’s *α* = .51–.80; see Appendix C). In the next step, the coding scheme was further refined through discussions between the two raters and the first author; because of good inter-rater agreement, the final coding was conducted by one of the raters. Subsequently, the outcome variable need-supportive communication strategy use was calculated using the sum of the applied SDT-based communication strategies for every measurement time point (preintervention, postintervention, and 1-week follow-up). At every time point, participants wrote 3 responses to postings (3 responses with up to 6 need-supportive communication strategies used per posting; possible range: 0–18 uses).

#### 2.3.2. Study 2

Demographic characteristics included age, gender, highest educational attainment, and highest professional degree, derived from the longitudinal German Internet Panel study (Blom, Gathmann, & Krieger, 2015, 2018). Educational attainment level was coded as in Study 1. Occupational skill level was coded according to the International Standard Classification of Occupations (ISCO; International Labour Office, 2012) as ISCO skill level 1 (low, e.g. unskilled worker), level 2 (medium, e.g. skilled worker), level 3 (high, e.g. higher skilled worker), and level 4 (very high, e.g. academic job). ISCO levels 3 and 4 were subsequently integrated into one category (high to very high).

Other control variables included if participants followed an omnivore diet (0 vs. 1) or a weight loss diet (0 vs. 1), if participants had a fructose intolerance (0 vs. 1), and the mean number of other active community members in the individual intervention period, which could have differed between the different online communities and recruitment phases.

##### 2.3.2.1. Primary outcomes

Perceived need support in the online community was measured with the 15-item Virtual Care Climate Questionnaire which is based on Self-Determination Theory and other established questionnaires for assessing perceived autonomy-support (Smit, Dima, Immerzeel, Putte, & Williams, 2017; example item: ‘The other forum users give me the feeling that I myself can choose a way to increase my TARGET BEHAVIOR by a third’; Cronbach’s *α* = .92). The questionnaire assesses perceived autonomy/ need-support in virtual settings (e.g. Smit et al., 2017).

Goal attainment scores were calculated by dividing the *values of the self-reported behaviors* at follow-up (e.g. number of vegetable portions or weekly minutes of moderate physical activity) by the behavioral goal (i.e. baseline behavior increased by 33%). A value of 1.0 thus represents 100% goal attainment.

Physical activity was measured (in minutes per week) with 2 items each for moderate physical activity and vigorous physical activity derived from the International Physical Activity Questionnaire Short Form (Craig et al., 2003; Kim, Park, & Kang, 2013). Example items for moderate physical activity include ‘During the last 7 days, on how many days did you do moderate physical activities (breathing and heartbeat are increased, speaking is still easy, but singing is no longer possible) like carrying light loads, riding a bicycle at normal speed, doing strenuous household chores, playing actively with children, or doing moderate-intensity sports and endurance sessions at home, in the gym, or out in the fresh air? Do not include walking’ and ‘How much time did you usually spend doing moderate physical activities on one of those days?’

Fruit and vegetable intake was measured with the 2 open-ended questions ‘How many daily portions of fruits/vegetables did you eat on average in the last 7 days?’ (cf. Chapman, Armitage, & Norman, 2009; Zhou, Gan, Hamilton, & Schwarzer, 2017) after receiving information about typical portion sizes according to the German Federal Centre for Nutrition and the ‘Five-a-Day’ campaign (5 am Tag e.V., 2014; German Federal Centre for Nutrition, 2020).

Engagement was measured with 3 indicators. The subjective forum visit frequency was measured with the item ‘How often did you visit the online forum during the challenge period?’ (response scale from 1 = not at all to 7 = multiple times daily). The number of logins and the number of postings within the 2-week intervention period were determined for each participant from the objective server data.

To examine whether the intervention had the hypothesized effect, we again calculated the need-supportive communication strategy use for every posting (possible range: 0–6) as an additional proximal outcome. Strategy use, along with other variables such as if a posting contained self-monitoring (0 vs. 1) or a problem description (0 vs. 1), was coded by 2 trained raters who also initially extended the coding manual of Study 1 in joint discussions. The raters were blind to the experimental condition. After coding and comparing 50 postings and the refinement of the initial coding manual, 10.24% (166/1621) of the postings were coded by both raters to calculate interrater agreement, which was good (percentage agreement = 91.0%–98.2%; Krippendorff’s *α* = .27–.92; see Appendix C). The rest of the postings were divided among the 2 raters who each coded half of the postings.

##### 2.3.2.2. Secondary outcomes

Perceived social support was measured with 9 items adapted from the Child and Adolescent Social Support Scale for Healthy Behaviors (Menon & Demaray, 2013), for example, ‘The other forum users encourage me to maintain or increase my current TARGET BEHAVIOR’; Cronbach’s *α* = .92.

Instrumental and experiential attitude were measured with 5 and 4 semantic differential scales, respectively, (Conner, Rhodes, Morris, McEachan, & Lawton, 2011; Lawton, Conner, & McEachan, 2009). Items include ‘For me, maintaining or increasing my TARGET BEHAVIOR would be: useless–useful’ and ‘For me, maintaining or increasing my TARGET BEHAVIOR would be: unpleasant–pleasant’; Cronbach’s *α* = .87 for each.

Self-efficacy was measured with 4 items adapted from the preaction and maintenance self-efficacy scales of the Health Action Process Approach measures (Schwarzer, 2007; Sniehotta, Scholz, & Schwarzer, 2005), for example, ‘I am sure I can maintain or increase my TARGET BEHAVIOR, even if I do not see success at once’; Cronbach’s *α* = .78.

Perceived descriptive and injunctive norms were measured with 3 items for each construct following the recommendations for social norm items of the Reasoned Action Approach (Fishbein & Ajzen, 2011), such as ‘I think most forum members intend to maintain or increase their TARGET BEHAVIOR’ and ‘I think most forum members expect me to maintain or increase my TARGET BEHAVIOR’; Cronbach’s *α* = .92 and .95.

Autonomous motivation and controlled motivation were measured with 10 items adapted from the Behavioral Regulation Sports Questionnaire (Lonsdale, Hodge, & Rose, 2008). Specifically, for each behavioral regulation form, two items with the highest item loadings were chosen and adapted to fit the target behavior. Subsequently, intrinsic motivation, integrated motivation, and identified motivation scores were combined to measure autonomous motivation; introjected motivation and external motivation scores were combined to measure controlled motivation. Example items include ‘I intend to maintain or increase my TARGET BEHAVIOR because it’s fun’ and ‘I intend to maintain or increase my TARGET BEHAVIOR because I feel pressure from other people to do so’; Cronbach’s *α* = .78 and .67.

### 2.4. Statistical analyses

Analyses of both studies were conducted with study completers (available follow-up data) using R version 4.0.1. To test the effects of the condition and time variables, lmerTest was used to estimate linear mixed models, and glmmADMB was used to estimate nonlinear mixed models. The R package emmeans was used to conduct Tukey-corrected post hoc contrasts with estimated marginal means. The R package performance was used to test for overdispersion and zero inflation in the nonlinear mixed models. The R package compareGroups was used to compare the different subgroups of the sample.

#### 2.4.1. Study 1

The condition variable (intervention vs. control) and the time variable (preintervention vs. postintervention vs. follow-up) were effect-coded, so main and interaction effects can be interpreted independently. The intervention condition’s and time variable’s effects on need-supportive communication strategy use were tested with a linear mixed model with the different measurement time points nested within persons and a random intercept on the person level. Analysis of variance type III tables were calculated with the R package car.

#### 2.4.2. Study 2

**Figure 2.**
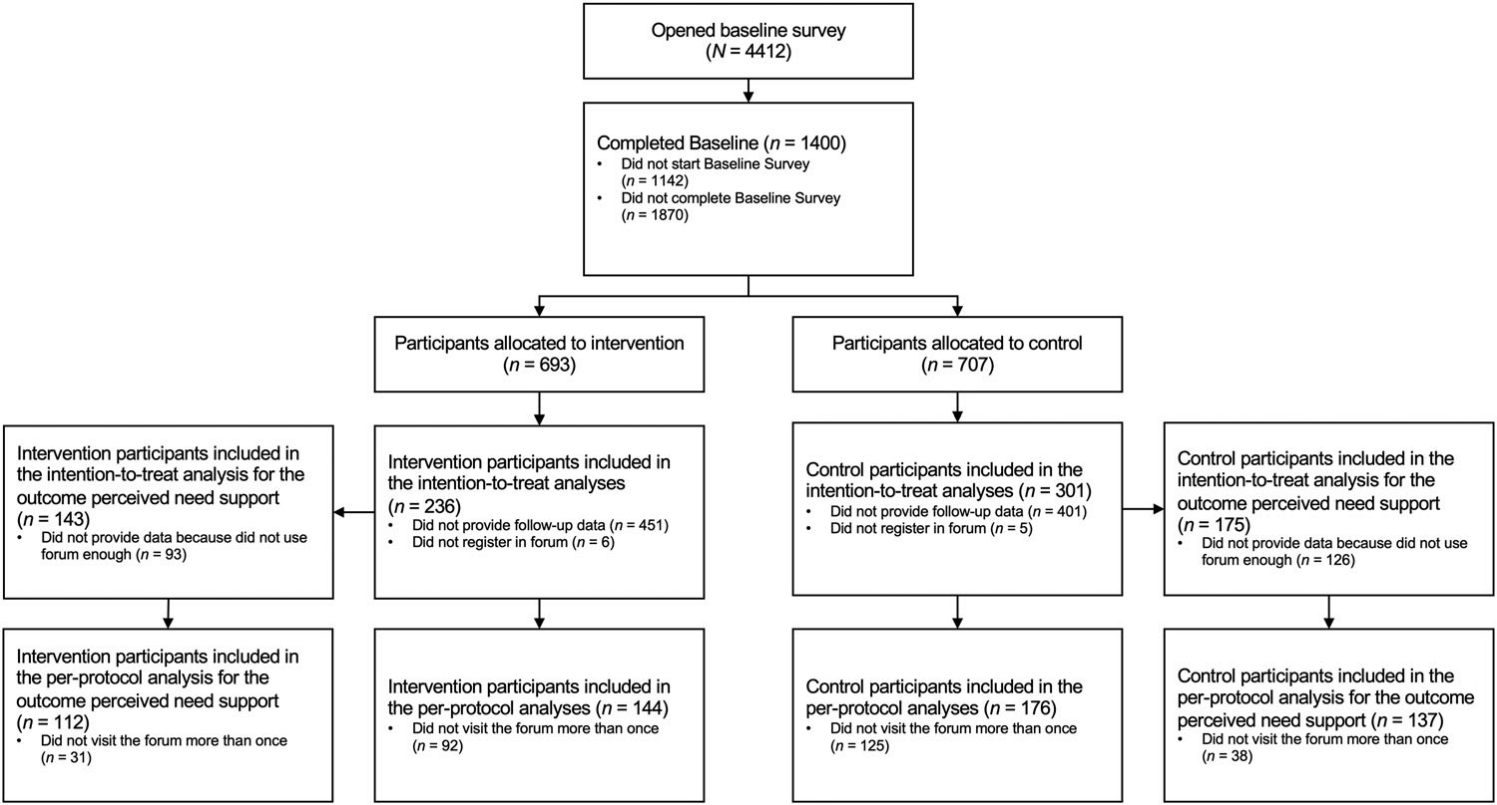
Participant flowchart for Study 2.

The condition variable was dummy coded in Study 2 to compare the two groups at follow-up directly. Outliers were handled using winsorization based on 3 times the median absolute deviation (Leys, Ley, Klein, Bernard, & Licata, 2013) and including them in the analyses for most outcomes to preserve statistical power (Leys, Delacre, Mora, Lakens, & Ley, 2019). We used the raw values for the count variables number of logins and number of postings because the data were highly skewed to the right and represented objective data points (see below for statistical models applied). All analyses were conducted twice, once using winsorized values and once excluding outliers. Instead of analyzing the engagement descriptively via the number of forum entries/ threads and number of responses per entry (as preregistered) we chose a more finely grained approach and analyzed the effect of the condition on the number of postings at the person level as an intensity measure of engagement (see also Short et al., 2018 for similar approaches). This decision was made to (1) increase the sample size for the analyses (there were only few entries, but many responses to entries) and to (2) account for the clustering of postings within participants and participants within the different forums. Additionally, we analyzed the number of logins and the subjective forum visit frequency as engagement measure. The number of logins can be seen as a frequency measure of engagement (Short et al., 2018) which we use as an indicator of the engagement of lurkers who do not post in the forums but may still benefit by passively viewing the content (Edelmann, 2016). We additionally analyzed the subjective forum visit frequency to see if the results for this more indirect form of engagement match in measures of both actual and perceived engagement. The intervention condition's effects on the outcomes were tested with linear mixed models for all primary and secondary outcome variables except for the number of logins and postings. The effect on the number of logins and postings was tested with negative-binomial-distributed mixed models because the variables represented counts and the presence of substantial overdispersion (Gelman & Hill, 2006). Additionally, we modeled zero inflation if detected (Gelman & Hill, 2006). As an additional proximal outcome, we estimated the intervention condition’s effect on the coded need-supportive communication strategy use on the posting level with a Poisson-distributed mixed model. The mixed models contained the maximal random effect structure justified by design to maximize generalizability and control type-I error rate (Barr, Levy, Scheepers, & Tily, 2013; Musca et al., 2011). That is, for the number of need-supportive communication strategies, we included random intercepts and random slopes (predictor: condition) for participants and forums to account for potential systematic differences in mean levels (random intercepts) and intervention-effects (random slopes) between participants and in the different forums because the observations (Level 1) were clustered within participants (Level 2) and forums (Level 3). For all other outcomes (e.g. number of logins and goal attainment), we included random intercepts and slopes (predictor: condition) for the cluster variable forum to account for potential systematic differences in mean levels (random intercepts) and intervention-effects (random slopes) in the different forums because participants (Level 1) were clustered within forums (Level 2). For most outcomes, the models did converge. We only excluded the random slope in the models for the outcomes perceived need-support and experiential attitude because of non-convergence. All models further included control variables (only variables where systematic differences between the two conditions or between the self-selected goal types were detected) and baseline values of the dependent variables (when available). To check the robustness of our results, the analyses were conducted with all participants who registered in the forum and provided follow-up data, regardless of whether they used the forum (following the intention-to-treat principle), as well as with participants who visited the forum more than once (per-protocol analyses; see the flowchart in Figure 2). With the per-protocol analyses we aimed to exploratorily examine if potential intervention effects would replicate in the smaller but less noisy sample of actual users of the online communities. The results of the intention-to-treat analyses with winsorized values are reported in the main text, tables, and figures, if not stated otherwise. We refrained from testing the expected indirect effects of the intervention video on behavior change because we did not find any effects on the expected mediators in the first place.

For sample size estimation in Study 2, we relied on previous short-term intervention studies targeting the provision of need support that suggested a medium to strong effect size on average; however, the lower level bound of the confidence interval implies that the effect size could also be small (Su & Reeve, 2011). We therefore conservatively estimated a required sample size of *N* = 292 (*n* = 146 in each experimental group) to detect small effect sizes with a power of .80 at an *α* level of .05 (see preregistration).

## 3. Results

### 3.1. Study 1

#### 3.1.1. Participants and descriptive statistics

A total of 99 participants completed the baseline questionnaire, of whom 77% (76/99) also completed the follow-up questionnaire after 1 week. There were no significant baseline differences in demographic characteristics between completers and noncompleters. Participants in the final data set were mostly female (59/99, 78%), had a mean age of *M* = 25.2 years (*SD* = 11.4 years), and had medium to high educational attainment. There were no significant baseline differences in demographic characteristics and need-supportive communication strategy use preintervention between the two experimental conditions (see Table 1).

**Table 1.** Baseline characteristics and participant differences in the control and intervention conditions (Study 1).

Variable	Control group (*n* = 36)	Intervention group (*n* = 40)	*p*
Demographic characteristics			
Age (years), *M* (*SD*)	26.00 (13.30)	24.52 (9.53)	.584
Female, *n* (%)	29 (81)	30 (75)	.594
Educational attainment, *n* (%)			
Low (ISCED 0–2)	0 (0)	0 (0)	.881
Medium (ISCED 3 & 4)	28 (78)	32 (80)	
High (ISCED 5–8)	7 (19)	8 (20)	
Other	1 (3)	0 (0)	
Outcome (preintervention)			
Need-supportive communication strategy use (number of need-supportive communication strategies), *M* (*SD*)	5.47 (3.1)	4.65 (2.51)	.211

Note: The control and intervention groups were compared with Welch’s two-sample *t* tests (means) or Fisher’s exact tests (proportions) whereby *p* shows the significance level of the comparisons. ISCED = International Standard Classification of Education.

#### 3.1.2. Need-supportive communication strategy use

**Figure 3.**
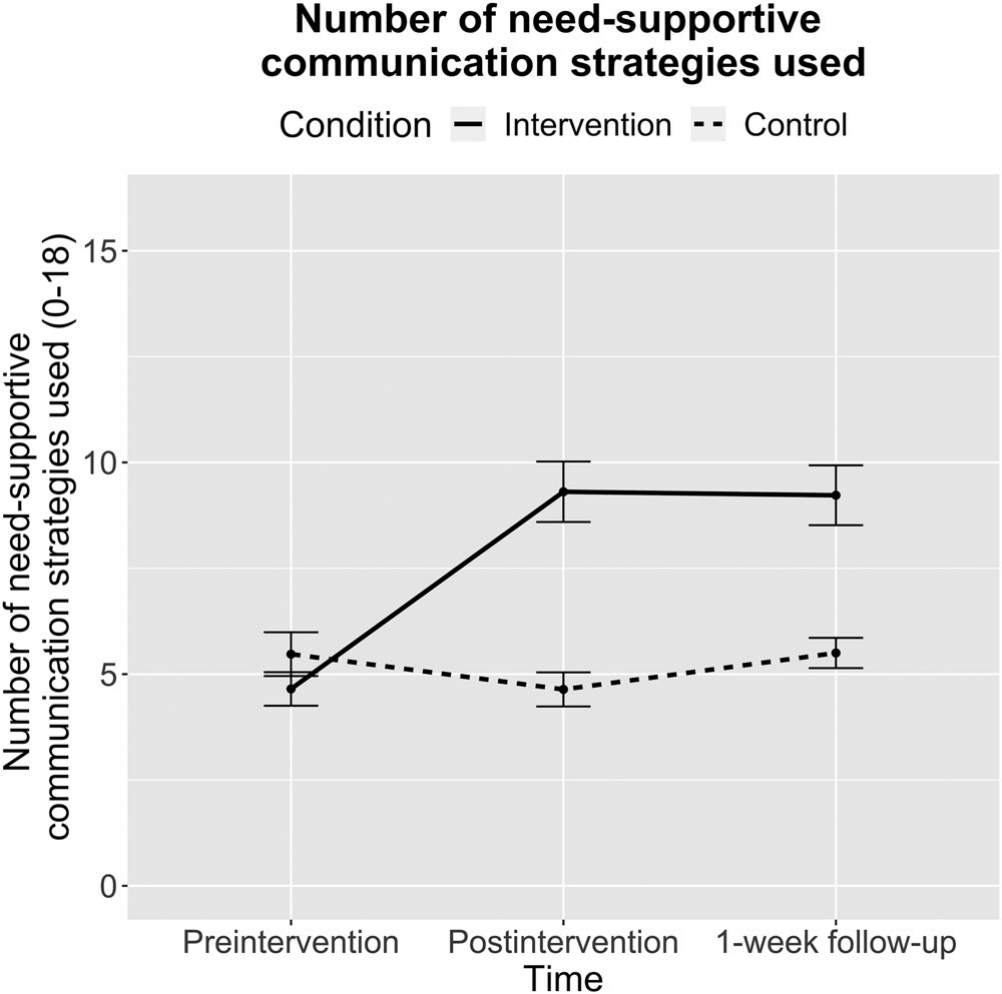
Mean number of need-supportive communication strategies used (Study 1). Note: Means and standard errors (error bars) for the number of need-supportive communication strategies used in the responses to fictive online postings, by measurement time and experimental condition.

For the outcome need-supportive communication strategy use, there was a significant main effect of condition (*F(1,* 72.92) = 14.91; *p *< .001; *partial η*^2^ = 0.17), a significant main effect of time (*F(2,* 148.17) = 23.75; *p *< .001; *partial η*^2^ = 0.24), and a significant interaction effect of condition × time (*F(2,* 148.17) = 33.75; *p *< .001; *partial η*^2^ = 0.31). Post hoc contrasts with Tukey adjustment showed that the number of need-supportive communication strategies used in the written responses increased in the intervention condition from preintervention to postintervention (estimate = –4.71, *SE* = 0.50; *t(*148) = –9.45; *p *< .001; Cohen’s *d *= –2.16, 95% CI [−2.91 to –1.41]) and from preintervention to follow-up (estimate = –4.58, *SE* = 0.50; *t(*148) = –9.27; *p *< .001; Cohen’s *d *= –2.10, 95% CI [–2.61 to –1.59]), but there were no significant changes in the control condition (all *p*s > .240). There was no difference in the mean number of need-supportive communication strategies used between the two groups at baseline (estimate = 0.83, *SE* = 0.78; *t(*132) = 1.07; *p *= .288; Cohen’s *d *= 0.38, 95% CI [–0.33–1.09]), but the intervention condition had a higher mean number of need-supportive communication strategies used postintervention (estimate = –4.71, *SE* = 0.78; *t(*133) = –6.05; *p *< .001; Cohen’s *d *= –2.16, 95% CI [–2.96 to –1.37]) and at 1-week follow-up (estimate = –3.72, *SE* = 0.78; *t(*132) = –4.80; *p *< .001; Cohen’s *d *= –1.71, 95% CI [–2.47 to –0.95]; for all means and standard errors, see Figure 3).

### 3.2. Study 2

#### 3.2.1. Participants and descriptive statistics

A total of 1400 participants completed the baseline assessment. After study dropout (lost to follow-up) and exclusion of 11 participants because they did not register in the forum, *n* = 537 participants could be included in the intention-to-treat analyses for most outcomes (537/1400, 38.36%). *N* = 320 participants could be included in the per-protocol analyses after excluding participants who did not log in more than once (320/1400, 22.86%; see participant flowchart in Figure 2).

There was a higher dropout in the intervention condition compared to the control condition (*OR* 0.70, 95% CI [0.56–0.87]; *p *= .001). Furthermore, study completers made on average more postings (*t(*637.95)_ _= –8.48; *p* < .001; Cohen’s *d *= 0.55, 95% CI [0.43–0.66]), logged in more often (*t(*648.01) = –8.64; *p *< .001; Cohen’s *d *= 0.56, 95% CI [0.44–0.67]), and had a lower fruit intake at baseline (*t(*1169.10)_ _= 2.04; *p *= .041; Cohen’s *d *= –0.11, 95% CI [–0.22–0.00]). There were no significant baseline differences in participant characteristics between the control and intervention group besides a lower vegetable intake at baseline in intervention participants, *t(*526.86) = 1.94; *p *= .048; Cohen’s *d *= –0.17, 95% CI [–0.34–0.00] (see Table 2). As would be expected, we found baseline differences between participants who had self-selected different behavioral goals. Therefore, we included these variables as control variables in the mixed models (see Appendix D for a detailed overview of baseline differences between the different goal-type conditions).

**Table 2.** Baseline characteristics and participant differences in the control and intervention group (Study 2).

Variable	Control group (*n* = 301)	Intervention group (*n* = 236)	*p*
Demographic characteristics			
Age (years), *M* (*SD*)	42.02 (11.64)	43.46 (11.30)	.150
Body mass index (kg/m^2^), *M* (*SD*)	28.52 (6.52)	28.57 (6.69)	.939
Female, *n* (%)	294 (97.7)	231 (97.9)	.999
Educational attainment, *n* (%)			.999
Low (ISCED 0–2)	3 (1.0)	2 (0.9)	
Medium (ISCED 3 & 4)	138 (45.9)	108 (45.8)	
High (ISCED 5–8)	159 (52.8)	126 (53.4)	
NA	1 (0.3)	0 (0.0)	
Occupational skill level, *n* (%)			.942
Low (ISCO skill level 1, e.g. unskilled worker)	24 (8.0)	16 (6.8)	
Medium (ISCO skill level 2, e.g. skilled worker)	117 (38.9)	92 (39.0)	
High (ISCO skill levels 3 & 4, e.g. higher skilled worker/academic job)	159 (52.8)	127 (53.8)	
NA	1 (0.3)	1 (0.4)	
Professional position, *n* (%)			.300
Full-time employees	152 (50.5)	108 (45.8)	
Part-time employees	74 (24.6)	73 (30.9)	
Students (higher education)	25 (8.3)	23 (9.8)	
Other	50 (16.6)	32 (13.6)	
Goal type, *n* (%)			.695
Fruit intake	20 (6.6)	15 (6.4)	
Vegetable intake	84 (27.9)	56 (23.7)	
Moderate physical activity	84 (27.9)	67 (28.4)	
Vigorous physical activity	113 (37.5)	98 (41.5)	
Outcomes (baseline)			
Fruit intake (no. portions), *M* (*SD*)	1.51 (1.01)	1.50 (0.94)	.957
Vegetable intake (no. portions), *M* (*SD*)	2.05 (1.41)	1.83 (1.25)	.048
Moderate physical activity (min/week), *M* (*SD*)	214.70 (193.09)	203.23 (192.77)	.494
Vigorous physical activity (min/week), *M* (*SD*)	33.19 (35.72)	32.56 (35.51)	.837
Autonomous motivation, *M* (*SD*)	3.94 (1.00)	3.85 (0.92)	.311
Controlled motivation, *M* (*SD*)	4.12 (1.52)	4.17 (1.51)	.720
Instrumental attitude, *M* (*SD*)	7.00 (0.00)	7.00 (0.00)	N/A^a^
Experiential attitude, *M* (*SD*)	5.50 (1.14)	5.55 (1.14)	.593
Self-efficacy, *M* (*SD*)	3.82 (0.67)	3.78 (0.68)	.582
Perceived descriptive norms, *M* (*SD*)	3.88 (0.68)	3.95 (0.68)	.219
Perceived injunctive norms, *M* (*SD*)	3.94 (0.78)	4.01 (0.80)	.271
Other			
Number of other active forum members, *M* (*SD*)	165.92 (93.77)	167.57 (95.09)	.841
Omnivore diet, *n* (%)	185 (61.5)	153 (64.8)	.472
Weight loss diet, *n* (%)	70 (23.3)	53 (22.5)	.837
Fructose intolerance, *n* (%)	288 (95.7)	226 (95.8)	.999

Note: The control and intervention groups were compared with Welch’s two-sample *t* tests (means) or Fisher’s exact tests (proportions), whereby *p* shows the significance of the comparisons. The outcomes goal attainment, perceived need support, perceived social support, number of logins, number of postings, and subjective forum use frequency are only available at follow-up. ISCED = International Standard Classification of Education. ISCO = International Standard Classification of Occupations. NA = Missing values. N/A = Not applicable.

^a^ Not applicable because the distribution of the variable was highly skewed and there was little variance; after winsorization, all participants scored the highest value on the scale (7).

In the two-week intervention period, there were *N* = 1130 postings in total in the forums. Participants created on average *M* = 2.07 (*SD* = 3.88) postings. One half of participants (273/537, 50.8%) posted at least once, and 31.7% (170/537) posted more than once (273/1400, 19.5% and 170/1400, 12.1% of initially randomized participants). Among all postings, 25.22% (285/1130) were categorized as self-monitoring postings, 32.57% (368/1130) contained a problem description by participants, 27.88% (315/1130) contained goal setting, and 17.96% (203/1130) contained a personal introduction. Participants logged in *M* = 3.54 (*SD* = 4.97) times on average. Most participants who registered in the forums (503/537, 93.67%) logged in at least once and around two third (320/537, 59.59%) logged in more than once.

#### 3.2.2. Proximal outcome: need-supportive communication strategy use

**Figure 4.**
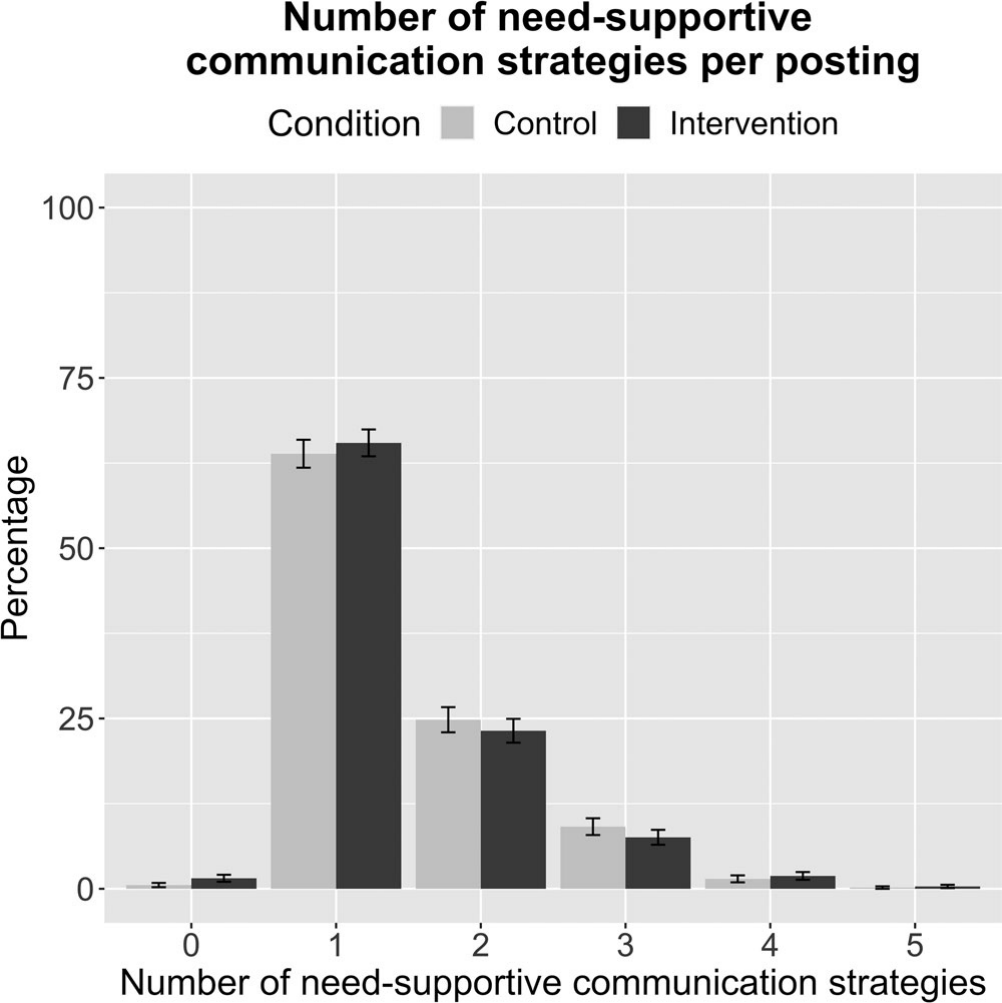
Percentage of postings with a specific number of need-supportive communication strategies based on Self-Determination Theory by experimental condition (Study 2). Note: The maximum possible number of strategies per posting is 6. Error bars represent 95% confidence intervals.

To examine whether our manipulation (i.e. watching the intervention or the control video) influenced the use of need-supportive communication strategies, we descriptively analyzed the proportion of postings with a specific number of strategies used per posting (maximum of 6 strategies per posting) in the *N* = 1130 included postings, separated by condition. As shown in Figure 4, most postings (63.87% (350/548) of participants’ postings in the control condition, 65.46% (381/582) of participants’ postings in the intervention condition) contained only one need-supportive communication strategy. Additionally, in the mixed model, there was no significant effect of condition on the number of applied strategies on the posting level (*B* = –0.01, *SE* = 0.05; *z *= –0.28; *p *= .778). This means that participants in the intervention condition did not use need-supportive communication strategies more frequently than participants in the control condition (*M*_Intervention_ = 1.44, *SD*_Intervention_ = 0.76; *M*_Control_ = 1.48, *SD*_Control _= 0.74). There were no intercept and slope variances (see Appendix E1), meaning that there were no differences in the mean levels of need-supportive communication strategy use between participants and the different forums. The effect of the video intervention did also not vary by participants and forums. The results did not change in the model with participants who visited the forum more than once.

#### 3.2.3. Primary outcomes

Not in line with the preregistered hypotheses, there were no statistically significant effects of the intervention video on perceived need support, goal attainment, and most of the engagement variables (see Table 3 for all primary and secondary outcomes). There was only a statistically significant effect of the intervention video on the number of postings (*B* = 0.31, *SE* = 0.15; *z *= 1.99; *p *= .046), as expected. More specifically, participants who watched the intervention video tended to have a higher number of postings compared to participants who watched the control video (see Table 3). Overall, there was little evidence for substantial slope variances for the effect of the video intervention on the primary outcomes (see Appendix E1), meaning that the effects did not vary between forums. For the outcomes ‘goal attainment’ and ‘subjective forum visit frequency’ there were also no substantial intercept variances, meaning that there were no systematic differences in the mean-levels of the outcome variables in the different forums. Most of the results did not change after excluding outliers based on the median absolute deviation or with participants who visited the forum more than once. One exception (when excluding outliers instead of winsorization) was a statistically significant effect of the intervention video on the subjective forum visit frequency (*B* = 0.28, *SE* = 0.10; *t(*514) = 2.71; *p *= .007). However, in participants visiting the forum more than once, the effect on subjective forum visit frequency was not statistically significant anymore (independent of the type of outlier handling). We also report sub-group analyses for the different health behaviors and goal types in Appendix F.

**Table 3.** Intervention effects and estimated marginal means for intervention and control condition from mixed models (Study 2).

Variable	Estimated marginal mean (*SE*)		Intervention effect estimate *B* (*SE*)^a^	*p*
	Control group (*n* = 301)	Intervention group (*n* = 236)		
Proximal outcomes				
Number of need-supportive communication strategies^b^	1.48 (0.03)	1.44 (0.03)	−0.01 (0.05)	.778
Primary outcomes				
Perceived need support	3.03 (0.16)	3.08 (0.17)	0.05 (0.22)	.840
Goal attainment	1.12 (0.07)	1.22 (0.07)	0.11 (0.09)	.260
Number of postings^b^	1.81 (0.20)	2.42 (0.28)	0.31 (0.15)	.046
Number of logins^b^	3.51 (0.33)	3.59 (0.25)	0.10 (0.08)	.250
Subjective forum visit frequency	2.47 (0.10)	2.71 (0.10)	0.24 (0.13)	.101
Secondary outcomes				
Autonomous motivation	3.99 (0.06)	3.96 (0.09)	−0.03 (0.10)	.795
Controlled motivation	4.20 (0.10)	4.09 (0.11)	–0.11 (0.13)	.384
Self-efficacy	3.39 (0.04)	3.39 (0.05)	–0.01 (0.06)	.915
Experiential attitude	5.13 (0.11)	5.20 (0.12)	0.07 (0.16)	.669
Instrumental attitude	6.58 (0.03)	6.64 (0.04)	0.06 (0.05)	.319
Perceived descriptive norms	3.77 (0.09)	3.77 (0.05)	0.00 (0.10)	.977
Perceived injunctive norms	3.38 (0.08)	3.47 (0.08)	0.09 (0.10)	.389
Perceived social support	3.23 (0.18)	3.28 (0.15)	0.05 (0.22)	.813

Note: Analyses were conducted with winsorized values and the effect of the intervention condition (dummy coded) is controlled for baseline values of the outcome, variables with baseline differences between completers and noncompleters, and between the 4 self-selected goal types, that is age, fruit intake, vegetable intake, moderate physical activity, vigorous physical activity, body mass index, perceived descriptive norms, perceived injunctive norms, and the mean number of active forum users. The raw values were used for the poisson-distributed count variables number of need-supportive communication strategies, number of logins and number of postings.

^a^ Differences between estimated marginal means and estimates originate from rounding.

^b^ For the number of postings and the number of logins, the intervention’s effect is multiplicative (*e*^estimate^) rather than additive since the models use a log-link-function for the count data. *M* (*SE*) represents the raw values because estimated marginal means could not be derived for nonlinear mixed models.

#### 3.2.4. Secondary outcomes

There were no statistically significant effects of the intervention video on autonomous motivation, controlled motivation, self-efficacy, perceived social support, experiential attitude, instrumental attitude, descriptive behavioral norms, or injunctive behavioral norms (see Table 3), which again was not in line the preregistered hypotheses. Overall, there were small to non-existent intercept and slope variances (see Appendix E2), meaning that the effect of the video intervention did not vary between the different forums, and that there were no systematic differences in the mean-levels of the outcome variables in the different forums. The results did not change when outliers were excluded based on median absolute deviation or in models including only participants who visited the forum more than once.

## 4. Discussion

### 4.1. Principal results

Consistent with our hypothesis, young adults who watched the intervention video with need-supportive communication strategies almost doubled their strategy use in written responses to fictive online postings immediately after watching the video and at 1-week follow-up in Study 1. In contrast, participants who watched a control video on netiquette rules showed no such increase directly after the intervention or at follow-up. These results suggest that a 3-minute educational video is sufficient to learn need-supportive communication strategies and apply them, at least for 1 week.

In Study 2, we tested whether this effect translates to a real-world online community, improving communication climate and perceived need support, using the same intervention videos. Not in line with our preregistered hypotheses, we found that participants in the intervention condition reported neither higher perceived need support from the other community members nor higher goal attainment compared to participants in the control condition. Goal attainment was generally very high: In both groups, participants reached on average more than 100% of their individual goals at the end of the intervention. We did only find mixed evidence regarding higher engagement in participants in the intervention condition. More specifically, they tended to have a higher perceived forum use (subjective forum visit frequency), but not actual forum use (number of logins); however, this effect was only inconsistent and not robust. Importantly, in line with our hypothesis, participants in the intervention condition showed a higher number of postings, compared to participants in the control condition. Not in line with our hypotheses, we did not find statistically significant differences between the two conditions at follow-up regarding behavior-related self-efficacy, experiential or instrumental attitudes, perceived descriptive or injunctive norms, perceived social support, or autonomous or controlled motivation. These findings are not surprising given that we expected the effects on our secondary outcomes to be mediated by changes in perceived need support (Hagger & Chatzisarantis, 2009), which we did not find in the first place. The results can be explained by a missing effect of the video intervention on the proximal outcome need-supportive communication strategy use.

Why did the strong increase in need-supportive communication strategy use not transfer from an experimental setting (Study 1) to a real-world setting (Study 2)? Regarding statistical power, we recruited an even higher sample size than preregistered (preregistered: *N* = 292 vs. final sample size: *N* = 537). This was the case because we conducted a community-based experiment where all participants had to start together. Participants had the chance to register for the study until two days before the intervention began; everyone who registered within this time window was allowed into the study. We did not shorten the envisioned time window to ensure enough participants at follow-up because we expected substantial study dropout (cf. Eysenbach, 2005). The larger sample size allowed us to detect small effects of the video intervention in Study 2 with a higher statistical power than initially targeted (.80 at an *α* level of .05). This was beneficial for our analyses because the effect size for our intervention had to be estimated on several theoretical assumption, because no similar intervention existed that could have informed this estimate. Additionally, Study 1 showed strong effects of the video intervention on need-supportive communication strategy use, suggesting that other reasons might underly the missing effects in Study 2. One reason could be that the strategies were not applicable to posting types that frequently occurred in the online communities. There was a substantial number of self-monitoring postings in which participants mainly tracked their goal progress without substantial or meaningful social interaction with other participants. The strategies, in contrast, aim to support other individuals who struggle with behavior change by fulfilling the basic psychological needs and enhancing the development of autonomous forms of motivation (Teixeira et al., 2020). Therefore, they are not applicable in self-monitoring posting. Further, all fictive online postings in Study 1 contained descriptions of barriers to a healthy diet, for which communication strategies such as ‘identifying problems and solutions’, as explained in the intervention video, were easily applicable. In contrast, less than a third of postings in Study 2 contained a problem description by participants to which all need-supportive communication strategies could theoretically be applied.

Additionally, goal attainment was very high in both groups, which is somewhat surprising because there is typically a so-called intention-behavior gap and people struggle with translating their intentions into behavior (Sheeran, 2002; Sheeran & Webb, 2016). Why might participants have been so successful in reaching their goals? There are at least two reasons which might explain the finding. First, our overall intervention contained effective BCTs (goal setting, provision of social support via forum) in both experimental groups, which supports the enaction of intentions into behavior. Furthermore, participants also used effective BCTs by themselves (e.g. self-monitoring, which was a frequently occurring posting type). Eating and physical activity interventions that include self-monitoring and other BCTs from control theory (e.g. goal setting) have been shown to be more effective than interventions who do not use them (Michie, Abraham, Whittington, McAteer, & Gupta, 2009). Second, the goals for participants were individualized, which make them more effective (Kwasnicka, Ntoumanis, & Sniehotta, 2021). Participants could choose their target behavior and the goal was adapted to the baseline level of the specific behavior. Research shows that goals are more effective when they are specific, personally relevant, and pursued for autonomous reasons (Kwasnicka et al., 2021). Because the intervention already included effective BCTs, participants might have not been in a high need for support by the other participants for very *specific* problems in the behavior change process – which was also reflected in the low number of postings containing a problem description. Taken together, the already successful behavior changes and high goal attainment in both groups may also partially explain the infrequent application of the need-supportive communication strategies, as they aim at supporting others who struggle with behavior change (Teixeira et al., 2020).

Lastly, another potential explanation for why the SDT-based video intervention did not produce the expected effects could be the generally low engagement in the online communities. Among participants who registered in the online community and provided follow-up data, one half contributed at least one post and around one third posted more than one. One reason for this could be that around half of the participants were also registered in at least one other online forum and around one third even actively participated – via reading and/or posting content – in other health-related online forums. This likely results in less time for using our study forum and might also reduce the need of participants to use our forum for the exchange and support with other people with shared goals. The relatively low engagement rate limits the impact of the need-supportive communication strategies on our hypothesized outcomes because they may simply not be applied frequently enough to change the perceived communication climate. Research suggests that a ‘critical mass’ is necessary to stimulate natural interaction within online communities and on social networking sites in general, which – despite some interaction among participants – may have been too low. There are usually many passive users or lurkers within online communities and on social networking sites (Carron-Arthur et al., 2015; Edelmann, 2016). Additionally, engagement rates in social-networking-site-based interventions typically vary substantially (Klassen et al., 2018; Waring et al., 2018; Williams et al., 2014). Research suggests that only about 10–20% of users in online communities are actively contributing (Edelmann, 2016). This means that general engagement rates in our study at around 30% of our follow-up sample posting more than once, and around 60% logging in more than once were still comparably high. In addition, having few very active users is also important: One study estimated that removing the 1% of very active superusers in two large online communities about asthma – who contributed about 32% and 49% of the postings – would cause the communities to collapse (Joglekar et al., 2018). Therefore, superusers keep online communities thriving. As discussed earlier, passive participation is not equal to nonuse or nonengagement. Lurkers may still profit from online communities and interventions by silently using the platform (Edelmann, 2016).

To sum up, high levels of goal attainment, low engagement, and low applicability of the need-supportive communication strategies to frequently occurring posting types might explain the missing effect of the intervention video on need-supportive communication strategy use. We expect that the effects of the video intervention might have been more pronounced if participants had shown stronger engagement and applied the need-supportive communication strategies more frequently in the interpersonal interactions. Interestingly, we found some evidence for an increase in engagement in participants watching the intervention video, even without increased need-supportive communication strategy use (increased number of postings). One possible explanation for this finding could be that the mere expectation of an improved communication climate in the online community stimulated engagement. Nevertheless, the effects were comparably small and might have been stronger and more reliable if the need-supportive communication strategies were applied more frequently in the interpersonal interactions.

### 4.2. Theoretical and practical implications

According to SDT (Deci & Ryan, 2000; Ryan et al., 2008; Ryan & Deci, 2017), a more need-supportive communication climate should lead to more autonomous and self-determined motivation, which should, in turn, lead to more effective behavior change. Additionally, it should positively influence behavior-related attitudes, perceived social norms, and self-efficacy, according to the integrated SDT model (Hagger & Chatzisarantis, 2009). Today, there is little research on need support in online communities and social networking sites. However, the results of previous offline randomized controlled trials show that health behavior interventions based on SDT can change perceived need support (Gillison et al., 2019; Ntoumanis et al., 2021). For example, in one SDT-based weight loss trial (Silva et al., 2008), providing a need-supportive environment led to higher perceived need support, autonomous motivation, behavior change, and weight loss, compared to a control group receiving a general health education curriculum (Silva et al., 2010, 2011). Interestingly, increased autonomous motivation for physical activity even spilled over to eating behavior regulation (Mata et al., 2009). Thus, increasing need support may even benefit health behaviors that are not directly targeted in an intervention. A recent meta-analysis showed that interventions to improve the provision of need support to others can be successful across different domains (Su & Reeve, 2011). It is important that future research further examines whether improving perceived need support in online communities and social networking sites leads to successful behavior change.

While short interventions can improve the provision of need support (Su & Reeve, 2011) and a short intervention format is generally promising for intervention delivery to large numbers of people (as typically found in online communities), short videos may not be intense enough to change a person’s communication style in a long-term field context. Therefore, future studies could test whether the incorporation of regular refreshers of intervention materials, such as watching the video again or short reminders about the key points, increases the expected effects. Yet another way to improve need-supportive communication strategy uptake and need-supportive communication could be to incentivize a proportion of the users for using the need-supportive communication strategies in their postings, making them peer role models. Relatedly, incentivizing participants for postings has been shown to increase engagement in a Facebook-delivered weight loss intervention (Pagoto et al., 2017). In addition, superusers or content moderators of online communities could receive more intense SDT-based training in need-supportive communication. For example, Inauen et al. (2017) trained and instructed confederate moderators in smartphone-based eating-related social support groups to model and ensure the provision of social support to participants by responding to every posting with supportive messages and posting daily questions.

### 4.3. Strengths and limitations

One limitation of Study 1 was the highly controlled experimental setting and a relatively homogeneous sample. While this can impact the ecological validity and generalizability of the findings, this controlled setting allowed us to test whether a short intervention video can change communication strategies under ideal conditions.

To address limitations inherent in a controlled experiment, we tested whether effects also applied in a real-world setting with a more heterogeneous sample (Study 2). While mostly women responded to our recruitment ads (e.g. print advertisement and Facebook ads), other characteristics such as age and body mass index substantially varied. Ecological validity was further increased by allowing participants to select their behavioral goals. This better reflects reality, where people usually choose to change a specific behavior, and it ensured that participants selected behavioral domains with room for improvement. The self-selection resulted on the other hand in unbalanced cell sizes for each behavior. In the analyses, we circumvented possible resulting power problems by pooling participants and focusing on goal attainment instead of the behavior itself in separate analyses. Nevertheless, this design feature also resulted in a different number of other active participants in the different forums. To account for this, we controlled for the mean number of other forum members in all analyses. To avoid such disbalance, future studies could focus on one behavior at a time (e.g. physical activity or vegetable intake) or include participants who choose different behaviors in the same forum. A more rigorous approach might be to randomize participants to the different goal behaviors. However, this could undermine participants’ motivation.

Study 2 participants had a comparably high rate of dropout to follow-up (863/1400, 61.6%). Surprisingly, there was a higher dropout in the intervention group compared to the control group. One potential reason for this is that it might have been more difficult for participants to apply the need-supportive communication strategies (compared to the more general netiquette rules) in the interpersonal communication, resulting in frustration and study dropout. This idea is indirectly supported by the fact that the use of need-supportive communication strategies was generally very low. While high dropout rates in general are not unusual for internet-based interventions (Eysenbach, 2005; Kelders, Kok, Ossebaard, & Gemert-Pijnen, 2012), the dropout in this study may have been influenced by a low adherence-motivating structure (e.g. without face-to-face appointments, reminders, or content moderators), no delivery of intervention content via the online communities, and the lack of a monetary incentive for study participation. Nevertheless, we recruited even more participants than preregistered, as discussed earlier. Because our intervention also aimed to increase engagement in the context of a mere community-support intervention, we did not include any additional strategies to target engagement. However, since engagement is typically low but important for intervention outcomes, the following strategies for enhancing engagement in interventions involving online communities or social networking sites in general could be helpful: including content moderators or (peer) role models (Inauen et al., 2017; Pagoto et al., 2017), increasing posting frequency, using call-to-actions in postings (Pagoto et al., 2016; Waring et al., 2018), using a combination of text, pictures, and videos in postings (Cavallo, Martinez, Webb Hooper, & Flocke, 2021; Waring et al., 2018), dependent on the possibilities of the specific platform, and also creating a positive and supportive communication climate, as we intended with our intervention.

One further limitation of Study 2 was the use of self-report questionnaires for fruit and vegetable intake and physical activity. For physical activity, assessments with questionnaires can result in lower reliability or validity and lead to systematic overestimation (Helmerhorst, Brage, Warren, Besson, & Ekelund, 2012; Prince et al., 2008) or underestimation of physical activity (Prince et al., 2008). Yet, self-reports with validated measures such as in our study are efficient, affordable, and can be easily administered in online forums. While accelerometers are not subject to recall bias, they can be very costly (Lee & Shiroma, 2014), not equally suitable for all kinds of activity (e.g. swimming, bicycling), and can produce different results depending on the model or how they are worn. Thus, they are difficult to use in an online-recruited study such as ours. Assessments of eating behavior such as food frequency questionnaires or 24-hour recall methods often rely on subjective measures that can lead to recall bias (Naska, Lagiou, & Lagiou, 2017). To improve reliability of the fruit and vegetable intake assessment in our study, we provided written examples of portion sizes and pictures. Importantly, self-report is the most feasible method in online studies. Nevertheless, future studies could consider new assessment methods such as photo-based recording (König, Van Emmenis, Nurmi, Kassavou, & Sutton, 2021) that may increase usability and reduce participant burden. Importantly, while absolute levels of target behaviors may be biased in our study, the goal attainment scores are informative, because both scores were measured with the same method within the same person, resulting in the same bias (e.g. systematic over- or underestimation) at every measurement point.

For Study 2, we created new online communities explicitly for our participants. Additional analyses, however, suggested that most participants who completed the follow-up questionnaire would prefer to join a private Facebook group (vs. a forum). When conducting interventions involving social networking sites, one of the big questions is whether to create a social networking site and online community from scratch or to use already established ones such as Facebook, Instagram, Twitter, or Reddit. In this decision, there is usually a trade-off between privacy and usability. Creating a social networking site from scratch for research purposes has the huge advantage that the data belongs to the researchers. There is no third-party company involved whose privacy guidelines must be applied. Furthermore, there is always full access to the necessary data. In addition, there are several challenges in using commercial applications for research (Arigo et al., 2019; Pagoto et al., 2016). For example, collected data typically belongs to the company and can be used for other purposes such as advertisement. It is essential to inform participants about the existing privacy guidelines and who has access to what data. Another challenge is limited data access for researchers, which could even change during the project because application programming interface permissions and restrictions frequently change. Advantages of commercial social networking site applications include their high usability, wide distribution, and integration into smartphones, thus allowing them to conveniently reach a large proportion of the population (Arigo et al., 2019). In line with this, one systematic review showed that interventions using private Facebook groups had the highest levels of engagement and acceptance (Klassen et al., 2018). Fortunately, there have been efforts to develop ethical standards for social networking site and social media research to protect user privacy, although they are still mostly uncoordinated (Arigo et al., 2019; Pagoto & Nebeker, 2019).

## 5. Conclusions

Social networking sites and online communities have a huge potential for supporting behavior change, but user engagement is typically low, and the quality of interpersonal communication and interaction needs to be improved to maximize effects. According to SDT, promoting need-supportive communication could positively influence both user engagement and behavior change. A brief video intervention could serve as a low-cost intervention to improve need-supportive communication. However, its applicability and effectiveness in more ecologically valid contexts need further evaluation. Complementary strategies such as training super-users or content moderators in need-supportive communication may improve strategy uptake and intervention effects. Future intervention studies should incorporate additional strategies for improving user engagement to further stimulate natural interpersonal interaction among users.

## Supplementary Material

Supplemental Material

## Data Availability

The data that support the findings of this study are openly available in the Open Science Framework at https://osf.io/xsb2e/.
