# Supplementary material for: A theory-based video intervention to enhance communication and engagement in online health communities: two experiments
Source: Health Psychol Behav Med. 2022 Feb 9;10(1):199–228. doi: 10.1080/21642850.2022.2032074 (PMC8843193; doi:10.1080/21642850.2022.2032074)
Supplement: Supplemental Material [file RHPB_A_2032074_SM4070.docx]

**Appendix**

**Appendix A: Randomization scheme and forum structure of the 4 online communities in Study 2.**


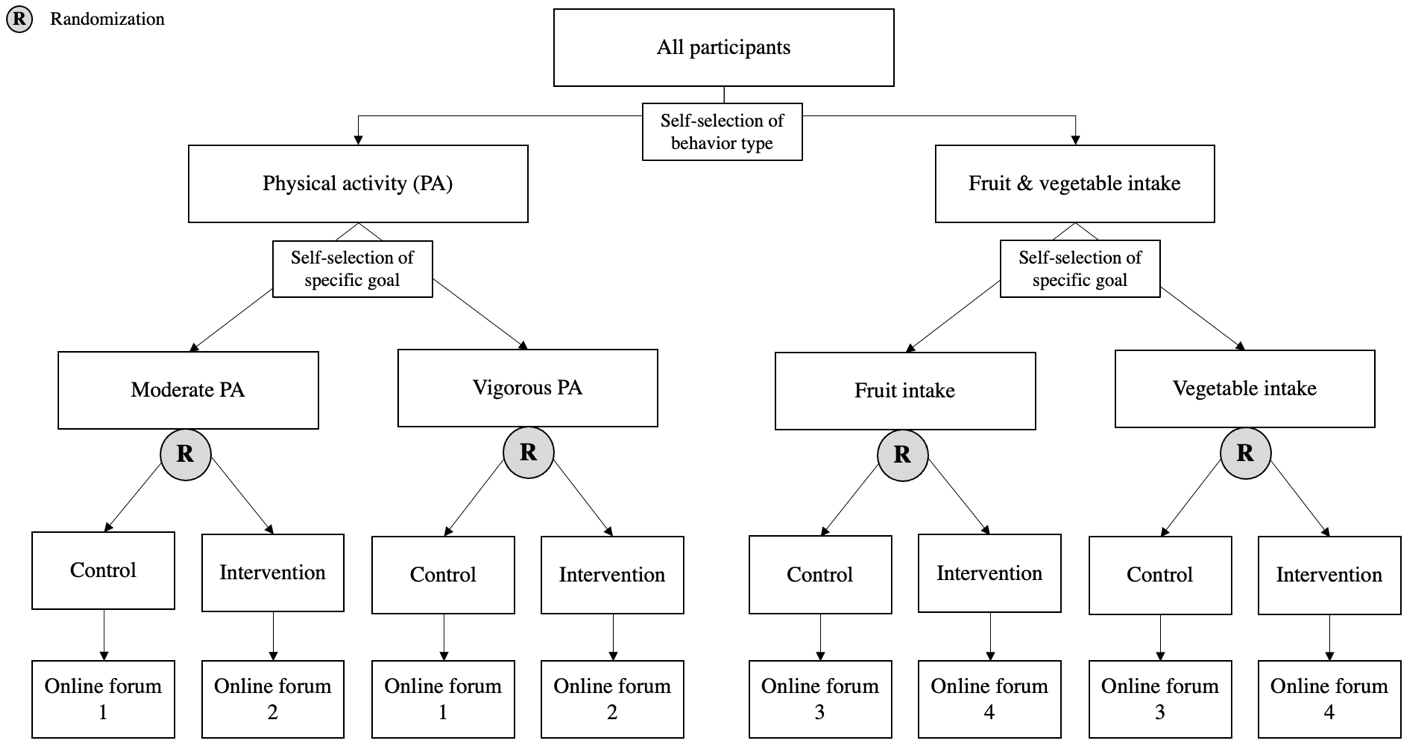


**
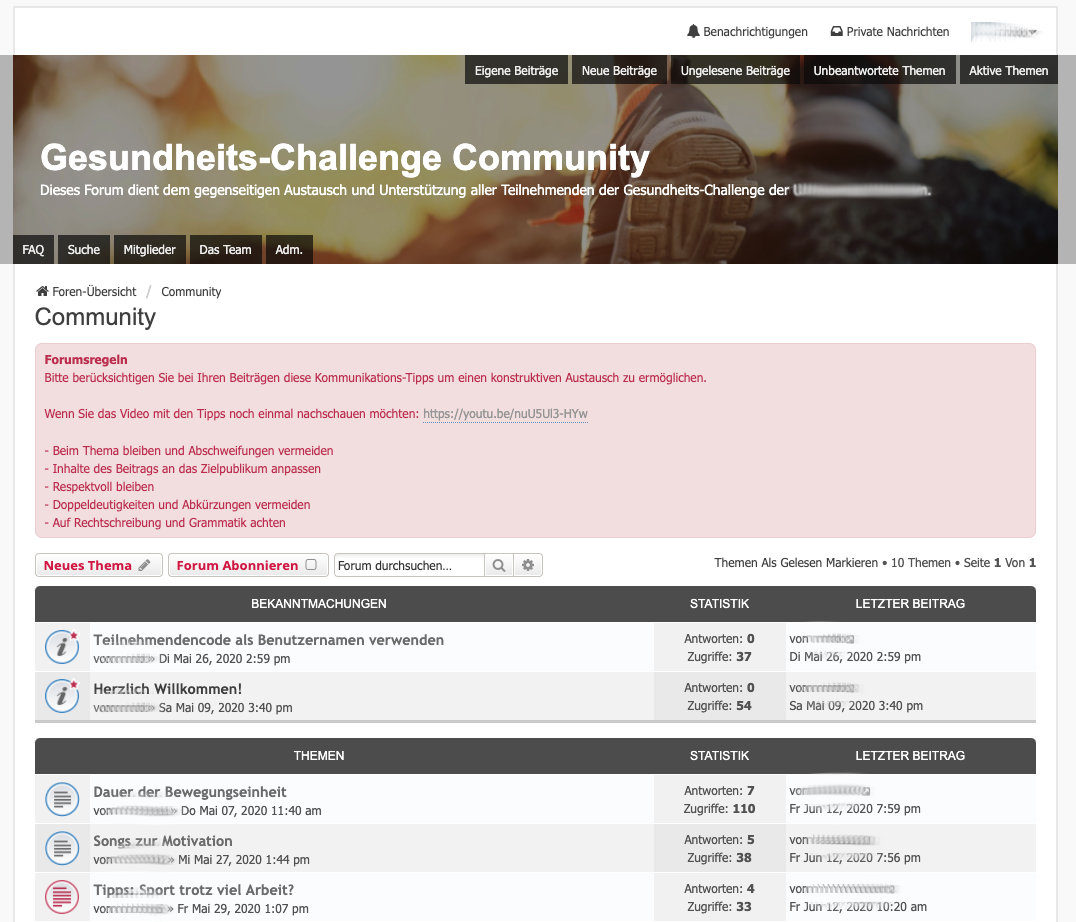
Appendix B: Screenshot of 1 of the 4 online communities (physical activity behavior and control condition) in Study 2.**

**Appendix C: Interrater agreement for variables in Study 1 and Study 2**

| **Table C1** | | | |
| --- | --- | --- | --- |
| *Interrater agreement for variables coded in written responses (Study 1) and postings of participants in the online communities (Study 2)* | | | |
| Variable | Agreement (%) | Krippendorff’s α^a^ | *N* |
| Study 1 |  |  |  |
| Providing options and offering choices | 90.6 | 0.80 | 170 |
| Using autonomy-supportive language | 76.5 | 0.51 | 170 |
| Acknowledging successes | 95.3 | 0.77 | 170 |
| Identifying barriers and solutions | 88.8 | 0.76 | 170 |
| Acknowledging negative and positive feelings | 78.2 | 0.56 | 170 |
| Offering contact | 94.1 | 0.79 | 170 |
| Study 2 |  |  |  |
| Providing options and offering choices | 96.4 | 0.80 | 166 |
| Using autonomy-supportive language | 93.4 | 0.39 | 166 |
| Acknowledging successes | 95.8 | 0.74 | 166 |
| Identifying barriers and solutions | 97.0 | 0.27 | 166 |
| Acknowledging negative and positive feelings | 94.0 | 0.82 | 166 |
| Offering contact | 96.4 | 0.77 | 166 |
| Providing solutions | 94.6 | 0.83 | 166 |
| Reference to other posting | 86.1 | 0.77 | 166 |
| Self-monitoring of behavior in posting | 94.6 | 0.87 | 166 |
| Goal setting in posting | 92.2 | 0.80 | 166 |
| Organizational posting | 98.2 | 0.74 | 166 |
| Introduction in posting | 97.0 | 0.92 | 166 |
| Problem description in posting | 91.0 | 0.81 | 166 |

*Note.* *N* denotes the number of postings in the calculation of the interrater agreement.

^a^Estimated population rate of observations (e.g., 1) based on the coded data influences Krippendorff α estimates, whereby higher rates punish Krippendorff’s α calculations because more agreements could be due to chance, which can lead to high percentage agreement but lower Krippendorff’s α estimates.

**Appendix D: Baseline characteristics and participant differences in the different self-selected goal types (Study 2).**

| **Table D1** | | | | | | |
| --- | --- | --- | --- | --- | --- | --- |
| *Baseline characteristics and participant differences in the different self-selected goal types (Study 2)* | | | | | | |
| Variable | Goal type | | | | *p* | Differences^a^ |
|  | Fruit intake  (FI; *n* = 35) | Vegetable intake (VI; *n* = 140) | Moderate physical activity (MPA; *n* = 151) | Vigorous physical activity (VPA; *n* = 211) |  |  |
| Demographic characteristics |  |  |  |  |  |  |
| Age (years), *M* (*SD*) | 43.11 (12.11) | 45.22 (10.51) | 44.10 (11.63) | 39.84 (11.41) | <.001 | VI > VPA  MPA > VPA |
| Body mass index (kg/m^2^), *M* (*SD*) | 27.89 (6.53) | 29.05 (6.29) | 30.63 (7.28) | 26.82 (5.79) | <.001 | VI > VPA  MPA > VPA |
| Female, *n* (%) | 35 (100.0) | 136 (97.1) | 147 (97.4) | 207 (98.1) | .383 |  |
| Educational attainment, *n* (%) |  |  |  |  | .518 |  |
| Low (ISCED 0–2) | 0 (0.0) | 2 (1.4) | 0 (0.0) | 3 (1.4) |  |  |
| Medium (ISCED 3 & 4) | 18 (51.4) | 60 (42.9) | 78 (51.7) | 90 (42.7) |  |  |
| High (ISCED 5–8) | 17 (48.6) | 78 (55.7) | 73 (48.3) | 117 (55.5) |  |  |
| NA | 0 (0.0) | 0 (0.0) | 0 (0.0) | 1 (0.5) |  |  |
| Occupational skill level, *n* (%) |  |  |  |  | N/A^b^ |  |
| Low (ISCO skill level 1, e.g., unskilled worker) | 2 (5.7) | 4 (2.9) | 15 (9.9) | 19 (9.0) |  |  |
| Medium (ISCO skill level 2, e.g., skilled worker) | 16 (45.7) | 58 (41.4) | 62 (41.1) | 73 (34.6) |  |  |
| High (ISCO skill level 3 & 4, e.g., higher skilled worker/academic job) | 17 (48.6) | 78 (55.7) | 73 (48.3) | 118 (55.9) |  |  |
| NA | 0 (0.0) | 0 (0.0) | 1 (0.7) | 1 (0.5) |  |  |
| Professional position, *n* (%) |  |  |  |  | N/A^b^ |  |
| Full-time employment | 15 (42.9) | 68 (48.6) | 77 (51.0) | 100 (47.4) |  |  |
| Part-time employment | 10 (28.6) | 41 (29.3) | 36 (23.8) | 60 (28.4) |  |  |
| Students (higher education) | 5 (14.3) | 9 (6.4) | 11 (7.3) | 23 (10.9) |  |  |
| Other | 5 (14.3) | 22 (15.7) | 27 (17.9) | 28 (13.3) |  |  |
| Outcomes (baseline) |  |  |  |  |  |  |
| Fruit intake (no. portions), *M* (*SD*) | 0.91 (0.82) | 1.37 (0.90) | 1.54 (0.96) | 1.67 (1.02) | <.001 | FI < MPA  FI < VPA  VI < VPA |
| Vegetable intake (no. portions), *M* (*SD*) | 1.48 (1.17) | 1.49 (1.13) | 2.15 (1.49) | 2.20 (1.31) | <.001 | FI < MPA  FI < VPA  VI < MPA  VI < VPA |
| Moderate physical activity (min/week), *M* (*SD*) | 203.89 (209.06) | 255.13 (199.84) | 144.59 (146.67) | 227.01 (203.29) | <.001 | VI >MPA  MPA < VPA |
| Vigorous physical activity (min/week), *M* (*SD*) | 34.90 (38.83) | 37.43 (36.81) | 22.71 (32.75) | 36.89 (34.95) | .001 | VI > MPA  MPA < VPA |
| Autonomous motivation, *M* (*SD*) | 3.76 (0.92) | 4.01 (0.87) | 3.91 (0.94) | 3.85 (1.05) | .350 |  |
| Controlled motivation, *M* (*SD*) | 3.98 (1.82) | 4.30 (1.61) | 4.01 (1.35) | 4.15 (1.51) | .385 |  |
| Instrumental attitude, *M* (*SD*) | 7.00 (0.00) | 7.00 (0.00) | 7.00 (0.00) | 7.00 (0.00) | N/A^c^ |  |
| Experiential attitude, *M* (*SD*) | 5.63 (1.07) | 5.67 (1.05) | 5.46 (1.23) | 5.44 (1.13) | .267 |  |
| Self-efficacy, *M* (*SD*) | 3.84 (0.73) | 3.86 (0.68) | 3.76 (0.67) | 3.78 (0.67) | .583 |  |
| Perceived descriptive norms, *M* (*SD*) | 3.76 (0.68) | 3.95 (0.68) | 4.03 (0.69) | 3.83 (0.66) | .015 | MPA > VPA |
| Perceived injunctive norms, *M* (*SD*) | 3.87 (0.75) | 4.02 (0.83) | 4.11 (0.79) | 3.85 (0.76) | .012 | MPA > VPA |
| Other |  |  |  |  |  |  |
| Number of other active community members, *M* (*SD*) | 94.47 (48.10) | 98.77 (44.88) | 198.45 (94.46) | 200.89 (93.56) | <.001 | FI < MPA  FI < VPA  VI < MPA  VI < VPA |
| Omnivore diet, *n* (%) | 27 (77.1) | 95 (67.9) | 85 (56.3) | 131 (62.1) | .060 |  |
| Weight-loss diet, *n* (%) | 4 (11.4) | 36 (25.7) | 42 (27.8) | 41 (19.4) | .080 |  |
| Fructose intolerance, *n* (%) | 0 (0.0) | 6 (4.3) | 10 (6.6) | 7 (3.3) | .309 |  |

*Note.* The 4 self-selected goal type conditions were compared with analyses of variance (means) or Fisher’s exact tests (proportions), whereby *P* shows the overall significance of the comparisons. ISCED = International Standard Classification of Education. ISCO = International Standard Classification of Occupations. NA = Missing values. N/A = Not applicable.

^a^The column Differences shows the statistically significant Tukey-adjusted pairwise contrasts.

^b^Not applicable because test statistic could not be calculated.

^c^Not applicable because the distribution of the variable was highly skewed and there was little variance; after winsorization, all participants scored the highest value on the scale (7).

**Appendix E: Full mixed models for the outcome variables in Study 2**

| **Table E1** | | | | | | | | | | | | | | | | |  |  |
| --- | --- | --- | --- | --- | --- | --- | --- | --- | --- | --- | --- | --- | --- | --- | --- | --- | --- | --- |
| *Mixed models for the primary outcomes of Study 2 at follow-up* | | | | | | | | | | | | | | | | |  |  |
| Effects | Number of need-supportive communication strategies^a^ | | | Perceived need-support | | | Goal attainment | | | Subjective forum visit frequency | | | Number of postings^a^ | | | Number of logins^a^ | | |
|  | *B* | *SE* | *p* | *B* | *SE* | *p* | *B* | *SE* | *p* | *B* | *SE* | *p* | *B* | *SE* | *p* | *B* | *SE* | *p* |
| Fixed effects |  |  |  |  |  |  |  |  |  |  |  |  |  |  |  |  |  |  |
| (Intercept) | 0.41 | 0.21 | .056 | 2.65 | 0.44 | <.001 | 1.43 | 0.32 | <.001 | 2.19 | 0.47 | <.001 | -0.34 | 0.65 | .599 | 0.70 | 0.34 | .040 |
| Condition (intervention) | -0.01 | 0.05 | .778 | 0.05 | 0.22 | .840 | 0.11 | 0.09 | .260 | 0.24 | 0.13 | .101 | 0.31 | 0.15 | .046 | 0.10 | 0.08 | .250 |
| Age | -0.01 | 0.00 | .035 | -0.01 | 0.00 | .045 | -0.00 | 0.00 | .320 | -0.00 | 0.01 | .857 | 0.01 | 0.01 | .328 | 0.00 | 0.00 | .962 |
| Body mass index | -0.00 | 0.00 | .861 | -0.01 | 0.01 | .250 | -0.00 | 0.01 | .538 | 0.01 | 0.01 | .285 | -0.01 | 0.01 | .691 | 0.00 | 0.01 | .880 |
| Fruit intake (baseline) | 0.01 | 0.03 | .831 | -0.02 | 0.06 | .706 | 0.04 | 0.04 | .369 | 0.00 | 0.07 | .998 | 0.01 | 0.08 | .915 | 0.08 | 0.05 | .101 |
| Vegetable intake (baseline) | 0.01 | 0.02 | .514 | -0.04 | 0.04 | .373 | 0.04 | 0.03 | .169 | -0.05 | 0.05 | .314 | -0.01 | 0.06 | .899 | -0.05 | 0.04 | .167 |
| Moderate physical activity | -0.00 | 0.00 | .895 | -0.00 | 0.00 | .411 | -0.00 | 0.00 | .380 | 0.00 | 0.00 | .587 | 0.00 | 0.00 | .716 | -0.00 | 0.00 | .603 |
| Vigorous physical activity (baseline) | 0.00 | 0.00 | .108 | 0.00 | 0.00 | .138 | -0.00 | 0.00 | .439 | 0.00 | 0.00 | .409 | 0.00 | 0.00 | .398 | 0.00 | 0.00 | .068 |
| Descriptive norms (baseline) | 0.03 | 0.05 | .591 | 0.18 | 0.10 | .081 | -0.08 | 0.07 | .266 | -0.18 | 0.11 | .099 | -0.22 | 0.16 | .162 | -0.03 | 0.08 | .725 |
| Injunctive norms (baseline) | -0.01 | 0.05 | .765 | 0.02 | 0.09 | .863 | 0.05 | 0.06 | .464 | 0.09 | 0.09 | .339 | 0.23 | 0.15 | .111 | 0.04 | 0.07 | .566 |
| Number of other active forum members | 0.00 | 0.00 | .142 | 0.00 | 0.00 | .169 | -0.00 | 0.00 | .961 | 0.00 | 0.00 | .003 | 0.00 | 0.00 | <.001 | 0.00 | 0.00 | <.001 |
| Random effects |  |  |  |  |  |  |  |  |  |  |  |  |  |  |  |  |  |  |
| Residual variance σ^2^ |  |  |  | 0.74 |  |  | 0.77 |  |  | 1.70 |  |  |  |  |  |  |  |  |
| Intercept variance _participant_ | 0.00 |  |  | - |  |  | - |  |  | - |  |  | - |  |  | - |  |  |
| Intercept variance _forum_ | 0.00 |  |  | 0.08 |  |  | 0.00 |  |  | 0.01 |  |  | 0.00 |  |  | 0.00 |  |  |
| Slope variance (condition) _forum_ | 0.00 |  |  | N/A^b^ |  |  | 0.00 |  |  | 0.01 |  |  | 0.00 |  |  | 0.00 |  |  |
| Slope variance (condition) _participant_ | 0.00 |  |  | - |  |  | - |  |  | - |  |  | - |  |  | - |  |  |
| *n* _forum_ | 8 |  |  | 8 |  |  | 8 |  |  | 8 |  |  | 8 |  |  | 8 |  |  |
| *n* _participant_ | 274 |  |  | 318 |  |  | 537 |  |  | 537 |  |  | 537 |  |  | 537 |  |  |
| *N* | 1130 |  |  | 318 |  |  | 537 |  |  | 537 |  |  | 537 |  |  | 537 |  |  |

*Note*. Analyses were conducted with winsorized values and the effect of the intervention condition (dummy coded) is controlled for baseline values of the outcome, variables with baseline differences between completers and noncompleters, and between the 4 self-selected goal types, that is age, fruit intake, vegetable intake, moderate physical activity, vigorous physical activity, body mass index, perceived descriptive norms, perceived injunctive norms, and the mean number of active forum users. The raw values were used for the poisson-distributed count variables number of logins and number of postings.

^a^For the number of need-supportive communication strategies, number of postings and the number of logins, the intervention's effect is multiplicative (e^estimate^) rather than additive since the models use a log-link-function for the count data. *M* (*SE*) represents the raw values because estimated marginal means could not be derived for nonlinear mixed models.

^b^N/A = not applicable (random effect could not be specified because of convergence problems). NA = not applicable.

| **Table E2** | | | | | | | | | | | | | | | | | | | | | | | | |
| --- | --- | --- | --- | --- | --- | --- | --- | --- | --- | --- | --- | --- | --- | --- | --- | --- | --- | --- | --- | --- | --- | --- | --- | --- |
| *Mixed models for the secondary outcomes of Study 2 at follow-up* | | | | | | | | | | | | | | | | | | | | | | | | |
| Effects | Autonomous motivation | | | Controlled motivation | | | Self-efficacy | | | Experiential attitude | | | Instrumental attitude | | | Perceived descriptive norms | | | Perceived injunctive norms | | | Perceived social support | | |
|  | *B* | *SE* | *p* | *B* | *SE* | *p* | *B* | *SE* | *p* | *B* | *SE* | *p* | *B* | *SE* | *p* | *B* | *SE* | *p* | *B* | *SE* | *p* | *B* | *SE* | *p* |
| Fixed effects |  |  |  |  |  |  |  |  |  |  |  |  |  |  |  |  |  |  |  |  |  |  |  |  |
| (Intercept) | 2.17 | 0.36 | <.001 | 2.72 | 0.56 | <.001 | 0.72 | 0.26 | .007 | 1.92 | 0.43 | <.001 | 6.09 | 0.16 | <.001 | 3.50 | 0.26 | <.001 | 2.56 | 0.36 | <.001 | 2.81 | 0.50 | <.001 |
| Condition (intervention) | -0.03 | 0.10 | .795 | -0.11 | 0.13 | .384 | -0.01 | 0.06 | .915 | 0.07 | 0.16 | .669 | 0.06 | 0.05 | .319 | 0.00 | 0.10 | .977 | 0.09 | 0.10 | .389 | 0.05 | 0.22 | .813 |
| Age | -0.00 | 0.00 | .226 | 0.00 | 0.01 | .512 | 0.00 | 0.00 | .642 | -0.01 | 0.00 | .239 | 0.00 | 0.00 | .040 | -0.00 | 0.00 | .416 | -0.01 | 0.00 | .024 | -0.01 | 0.01 | .018 |
| Body mass index | -0.01 | 0.01 | .300 | -0.02 | 0.01 | .100 | 0.01 | 0.00 | .029 | -0.00 | 0.01 | .610 | -0.00 | 0.00 | .414 | -0.00 | 0.00 | .661 | 0.01 | 0.01 | .186 | -0.01 | 0.01 | .342 |
| Fruit intake (baseline) | -0.00 | 0.05 | .918 | 0.05 | 0.07 | .548 | -0.01 | 0.03 | .672 | 0.07 | 0.05 | .196 | 0.00 | 0.02 | .897 | 0.05 | 0.04 | .168 | 0.02 | 0.05 | .745 | -0.02 | 0.07 | .763 |
| Vegetable intake (baseline) | 0.01 | 0.04 | .678 | 0.00 | 0.06 | .999 | 0.05 | 0.03 | .041 | 0.04 | 0.04 | .328 | 0.02 | 0.02 | .273 | -0.03 | 0.03 | .196 | 0.04 | 0.04 | .285 | -0.01 | 0.05 | .853 |
| Moderate physical activity (baseline) | -0.00 | 0.00 | .126 | -0.00 | 0.00 | .562 | 0.00 | 0.00 | .471 | 0.00 | 0.00 | .320 | -0.00 | 0.00 | .478 | -0.00 | 0.00 | .073 | -0.00 | 0.00 | .797 | -0.00 | 0.00 | .424 |
| Vigorous physical activity (baseline) | 0.00 | 0.00 | .434 | 0.00 | 0.00 | .132 | 0.00 | 0.00 | <.001 | 0.01 | 0.00 | <.001 | 0.00 | 0.00 | .018 | 0.00 | 0.00 | .447 | 0.00 | 0.00 | .137 | 0.00 | 0.00 | .291 |
| Perceived descriptive norms (baseline) | 0.20 | 0.08 | .015 | -0.09 | 0.13 | .477 | 0.04 | 0.06 | .536 | 0.03 | 0.09 | .755 | 0.05 | 0.04 | .166 | 0.02 | 0.06 | .690 | -0.12 | 0.09 | .180 | 0.15 | 0.11 | .179 |
| Perceived injunctive norms (baseline) | -0.02 | 0.07 | .763 | 0.05 | 0.11 | .623 | 0.06 | 0.05 | .213 | -0.01 | 0.08 | .932 | 0.04 | 0.03 | .218 | 0.11 | 0.05 | .026 | 0.34 | 0.07 | <.001 | 0.05 | 0.10 | .614 |
| Number of other active forum members | -0.00 | 0.00 | .917 | -0.00 | 0.00 | .706 | -0.00 | 0.00 | .554 | -0.00 | 0.00 | .204 | -0.00 | 0.00 | .529 | -0.00 | 0.00 | .211 | -0.00 | 0.00 | 0.180 | 0.00 | 0.00 | .101 |
| Autonomous motivation (baseline) | 0.40 | 0.04 | <.001 |  |  |  |  |  |  |  |  |  |  |  |  |  |  |  |  |  |  |  |  |  |
| Controlled motivation (baseline) |  |  |  | 0.45 | 0.04 | <.001 |  |  |  |  |  |  |  |  |  |  |  |  |  |  |  |  |  |  |
| Self-efficacy (baseline) |  |  |  |  |  |  | 0.46 | 0.05 | <.001 |  |  |  |  |  |  |  |  |  |  |  |  |  |  |  |
| Experiential attitude (baseline) |  |  |  |  |  |  |  |  |  | 0.58 | 0.04 | <.001 |  |  |  |  |  |  |  |  |  |  |  |  |
| Instrumental attitude (baseline) |  |  |  |  |  |  |  |  |  |  |  |  | NA^b^ |  |  |  |  |  |  |  |  |  |  |  |
| Random effects |  |  |  |  |  |  |  |  |  |  |  |  |  |  |  |  |  |  |  |  |  |  |  |  |
| Residual variance σ^2^ | 0.91 |  |  | 2.24 |  |  | 0.46 |  |  | 1.12 |  |  | 0.20 |  |  | 0.49 |  |  | 1.03 |  |  | 0.88 |  |  |
| Intercept variance _forum_ | 0.00 |  |  | 0.00 |  |  | 0.00 |  |  | 0.03 |  |  | 0.00 |  |  | 0.02 |  |  | 0.01 |  |  | 0.09 |  |  |
| Slope variance (condition) _forum_ | 0.01 |  |  | 0.00 |  |  | 0.00 |  |  | N/A^a^ |  |  | 0.00 |  |  | 0.02 |  |  | 0.01 |  |  | 0.06 |  |  |
| *n* _forum_ | 8 |  |  | 8 |  |  | 8 |  |  | 8 |  |  | 8 |  |  | 8 |  |  | 8 |  |  | 8 |  |  |
| *N* | 537 |  |  | 537 |  |  | 537 |  |  | 537 |  |  | 537 |  |  | 537 |  |  | 537 |  |  | 290 |  |  |

*Note*. Analyses were conducted with winsorized values and the effect of the intervention condition (dummy coded) is controlled for baseline values of the outcome, variables with baseline differences between completers and noncompleters, and between the 4 self-selected goal types, that is age, fruit intake, vegetable intake, moderate physical activity, vigorous physical activity, body mass index, perceived descriptive norms, perceived injunctive norms, and the mean number of active forum users. The raw values were used for the poisson-distributed count variables number of need-supportive communication strategies, number of logins and number of postings.
^a^N/A = not applicable (random effect could not be specified because of convergence problems).

^b^NA = not available because predictor was dropped from the model because of rank deficiency.

**Appendix F: Intervention effects on the different health behaviors separately for the different goal types**

| **Table F1** | | | | |
| --- | --- | --- | --- | --- |
| *Intervention effects on health behaviors and estimated marginal means for the intervention and control condition from mixed models (Study 2)* | | | | |
| Variable | Estimated marginal mean (*SE*) | | Intervention effect  estimate *B* (*SE*)^a^ | *p* |
|  | Control group | Intervention group |  |  |
| Health behavior |  |  |  |  |
| Fruit intake, no. of portions (*n* = 35) | 1.67 (0.31) | 2.39 (0.89) | ﻿0.72 (0.94) | .564 |
| Vegetable intake, no. of portions (*n* = 140) | 1.95 (0.13) | 2.37 (0.14) | 0.42 (0.15) | .005 |
| Moderate physical activity, min/week (*n* = 151) | 204.40 (24.08) | 214.79 (22.09) | 10.39 (31.41) | .760 |
| Vigorous physical activity, min/week (*n* = 211) | 105.21 (8.62) | 105.10 (9.26) | -0.11 (11.67) | .993 |

*Note.* Analyses were conducted with winsorized values and the effect of the intervention condition (dummy coded) is controlled for baseline values of the outcome and variables with baseline differences between both conditions, that is controlled motivation for fruit intake and moderate physical activity, and perceived descriptive and perceived injunctive norms for moderate physical activity.

^a^Differences between estimated marginal means and estimates originate from rounding.

| **Table F2** | | | | | | | | | | | | |
| --- | --- | --- | --- | --- | --- | --- | --- | --- | --- | --- | --- | --- |
| *Mixed models for different health behaviors at follow-up separately by goal type (sub-group analysis)* | | | | | | | | | | | | |
| Effects | Fruit intake | | | Vegetable intake | | | Moderate physical activity | | | Vigorous physical activity | | |
|  | *B* | *SE* | *p* | *B* | *SE* | *p* | *B* | *SE* | *p* | *B* | *SE* | *p* |
| Fixed effects |  |  |  |  |  |  |  |  |  |  |  |  |
| (Intercept) | 0.09 | 0.54 | .869 | 1.03 | 0.13 | <.001 | 108.66 | 94.66 | .254 | 54.04 | 9.99 | <.001 |
| Condition (intervention) | 0.72 | 0.94 | .564 | 0.42 | 0.15 | .005 | 10.39 | 31.41 | .760 | -0.11 | 11.67 | .993 |
| Fruit intake (baseline) | 0.80 | 0.17 | <.001 |  |  |  |  |  |  |  |  |  |
| Vegetable intake (baseline) |  |  |  | 0.62 | 0.07 | <.001 |  |  |  |  |  |  |
| Moderate physical activity (baseline) |  |  |  |  |  |  | 0.75 | 0.09 | <.001 |  |  |  |
| Vigorous physical activity (baseline) |  |  |  |  |  |  |  |  |  | 1.39 | 0.17 | <.001 |
| Controlled motivation (baseline) | 0.22 | 0.09 | .021 |  |  |  | 2.92 | 10.55 | .782 |  |  |  |
| Perceived descriptive norms (baseline) |  |  |  |  |  |  | -13.36 | 24.86 | .592 |  |  |  |
| Perceived injunctive norms (baseline) |  |  |  |  |  |  | 7.27 | 21.60 | .737 |  |  |  |
| Random effects |  |  |  |  |  |  |  |  |  |  |  |  |
| Residual variance σ^2^ | 0.67 |  |  | 0.73 |  |  | 28487.9 |  |  | 7142 |  |  |
| Intercept variance _forum_ | 0.08 |  |  | 0.00 |  |  | 274.2 |  |  | 0.00 |  |  |
| Slope variance (condition) _forum_ | 0.94 |  |  | 0.00 |  |  | 274.2 |  |  | N/A^a^ |  |  |
| *n* _forum_ | 4 |  |  | 4 |  |  | 4 |  |  | 4 |  |  |
| *N* | 35 |  |  | 140 |  |  | 151 |  |  | 211 |  |  |

*Note*. Analyses were conducted with winsorized values and the effect of the intervention condition (dummy coded) is controlled for baseline values of the outcome and variables with baseline differences between both conditions, that is controlled motivation for fruit intake and moderate physical activity, and perceived descriptive and perceived injunctive norms for moderate physical activity.

^a^N/A = not applicable (random effect could not be specified because of convergence problems).
